# A high-resolution genomic composition-based method with the ability to distinguish similar bacterial organisms

**DOI:** 10.1186/s12864-019-6119-x

**Published:** 2019-10-21

**Authors:** Yizhuang Zhou, Wenting Zhang, Huixian Wu, Kai Huang, Junfei Jin

**Affiliations:** 1 Laboratory of Hepatobiliary and Pancreatic Surgery, The Affiliated Hospital of Guilin Medical University, Guilin, Guangxi 541001 People’s Republic of China; 2 Peking-Tsinghua Center for Life Science, Academy for Advanced Interdisciplinary Studies, Peking University, Beijing, 100871 People’s Republic of China; 3 China-USA Lipids in Health and Disease Research Center, Guilin Medical University, Guilin, Guangxi 541001 People’s Republic of China; 4 Guangxi Key Laboratory of Molecular Medicine in Liver Injury and Repair, Guilin Medical University, Guilin, Guangxi 541001 People’s Republic of China

**Keywords:** Composition, Species delineation, Metagenomic binning, Strain typing, TETRA, TZMD, Taxonomy

## Abstract

**Background:**

Genomic composition has been found to be species specific and is used to differentiate bacterial species. To date, almost no published composition-based approaches are able to distinguish between most closely related organisms, including intra-genus species and intra-species strains. Thus, it is necessary to develop a novel approach to address this problem.

**Results:**

Here, we initially determine that the “tetranucleotide-derived z-value Pearson correlation coefficient” (TETRA) approach is representative of other published statistical methods. Then, we devise a novel method called “**T**etranucleotide-derived **Z**-value **M**anhattan **D**istance” (TZMD) and compare it with the TETRA approach. Our results show that TZMD reflects the maximal genome difference, while TETRA does not in most conditions, demonstrating in theory that TZMD provides improved resolution. Additionally, our analysis of real data shows that TZMD improves species differentiation and clearly differentiates similar organisms, including similar species belonging to the same genospecies, subspecies and intraspecific strains, most of which cannot be distinguished by TETRA. Furthermore, TZMD is able to determine clonal strains with the TZMD = 0 criterion, which intrinsically encompasses identical composition, high average nucleotide identity and high percentage of shared genomes.

**Conclusions:**

Our extensive assessment demonstrates that TZMD has high resolution. This study is the first to propose a composition-based method for differentiating bacteria at the strain level and to demonstrate that composition is also strain specific. TZMD is a powerful tool and the first easy-to-use approach for differentiating clonal and non-clonal strains. Therefore, as the first composition-based algorithm for strain typing, TZMD will facilitate bacterial studies in the future.

## Background

Genomic composition refers to a set of short oligonucleotide frequencies in a genome. It can be profiled as short oligonucleotides from two to nine nucleotides [1], especially dinucleotides [2], trinucleotides [3, 4] and tetranucleotides [5]. The GC content and codon bias, which are represented by dinucleotides and trinucleotides respectively, are two examples of genomic composition. Genomic composition has been extensively studied by nearest-neighbor frequency analysis [6–8], chaos game representation [4, 9, 10], and statistical methods such as the odds ratio for dinucleotide bias [11], the Codon Adaptation Index for codon bias [12], and relative abundance measures for trinucleotides and tetranucleotides [13]. Studies show that intragenomic composition is fairly constant [5, 14, 15], even in ameliorated horizontally transferred regions [16]. Additionally, it has been reported that closely related organisms show more similar compositions than distantly related organisms, and thus, composition has been used to infer phylogenies [17, 18]. Furthermore, intragenomic composition is generally more homogenous than intergenomic composition [2, 9, 19–21], even between species sharing environmental pressures and interactions [22], implying that composition is species specific [14]. Thus, genomic composition is also coined genomic signature [2, 14]. The greater homogeneity of intragenomic composition may possibly result from (but is not limited to) species-specific properties of replication and repair machineries [2, 20]. However, whether composition is also strain specific remains unknown.

As composition is species specific, it has been widely used for species differentiation [23, 24] and metagenomic binning (classifying sequences into species-level groups) [25–31]. Additionally, composition has been used to detect foreign sequences, including laterally transferred genes [32–35], phage and viral genomes [36–39], and plasmids [40, 41]. Although methods based on composition can distinguish most species [24], they still cannot distinguish some similar species [23, 24]. In addition, they cannot differentiate most intraspecific strains [23]. Thus, it would be very useful to develop a novel method with the ability to distinguish similar organisms, including similar species and intraspecific strains.

The ability to distinguish genomic composition increases with oligonucleotide size [1, 5, 14, 42]. However, the computing cost also correspondingly increases. To balance the distinguishing ability and computing cost, tetranucleotides are thus widely used [17, 23, 24, 35, 41, 43, 44]. To our knowledge, four statistical methods have been published for tetranucleotide profiling. All of these methods use the Pearson correlation coefficient to assess composition similarity [5, 42]. Here, we found that the tetranucleotide-derived z-value Pearson correlation coefficient (TETRA) method could represent the three other statistical methods and thus could be used as the reference method for comparison when developing powerful methods. Subsequently, we proposed the tetranucleotide-derived z-value Manhattan distance (TZMD) approach, which uses the Manhattan distance rather than the Pearson correlation coefficient to quantify composition differences, and demonstrated that genomic composition is also strain specific. Our results clearly showed that TZMD is a high-resolution method that provides slightly improved results for species differentiation and can distinguish similar organisms, including closely related species at the species level and subspecies or intraspecific strains below the species level. Most importantly, TZMD is the first genomic composition-based method to differentiate clonal and non-clonal strains. Thus, we anticipate that TZMD will be used for species differentiation or for strain typing to facilitate bacterial studies.

## Results

### Comparison of four published statistical methods

To date, four different statistical methods have been published to measure tetranucleotide usage biases from their expectations. The zero-order Markov method removes mononucleotide frequency biases under a random mononucleotide distribution to measure tetranucleotide frequency biases [17, 42]. The maximal-order Markov method removes component biases to calculate tetranucleotide usage biases [17, 42]. The z-value method, in addition to the maximal-order Markov model, takes tetranucleotide variances into account to measure tetranucleotide usage biases as z-values [5]. The relative tetranucleotide frequency method factors out all lower-order biases to determine tetranucleotide usage biases [20]. Then, tetranucleotide usage biases calculated with all these methods are subjected to Pearson correlation coefficient calculations to measure the composition similarity between two sequences [5, 42].

To compare the four statistical methods, we used 1779 queries (Additional file 1: Table S1) against 264 references (Additional file 1: Table S2) comprising 1964 intraspecific and 467,692 interspecific pairs to profile the tetranucleotide usage biases and then calculated the Pearson correlation coefficients. Because the composition is species specific [2, 9, 14, 19–21], an effective method should strongly reflect this feature. Therefore, we first determined the optimal Pearson correlation coefficient cutoffs for species differentiation for these methods. The *F*-score, which was previously applied to determine the optimal sequence similarity thresholds for 40 single-copy phylogenetic marker genes [45] and 16S rRNA genes [46] for species delineation of prokaryotes, was applied to determine the optimal cutoff with the highest *F*-score for species differentiation for each method. For the statistical test, we randomly sampled 200 distinct intraspecific pairs and 50,000 distinct interspecific pairs 10 times for each sampling. We found that the optimal cutoffs for all these methods were identical at 0.99 or 1.00 (Additional file 2: Figure S1). Our paired t-test showed that the z-value method generated significantly higher *F*-scores than the zero-order Markov method but similar *F*-scores as the maximal-order Markov method and the relative tetranucleotide frequency method (Additional file 2: Figure S2). These results were further confirmed using the Rand index (Additional file 2: Figure S3 and S4). In this context, TETRA, which calculates the Pearson correlation coefficient for z-values obtained by the z-value method, can be used as a representative method for the three other published methods. Therefore, in this study, we only compared TZMD with TETRA to show the high resolution of TZMD. In addition, our further analysis showed that the vast majority of intraspecific pairs with a Pearson correlation coefficient > 0.99 cutoff had a Pearson correlation coefficient value of 1.00 for all four methods (Additional file 2: Figure S5), indicating that they have no ability to distinguish most intraspecific strains. Accordingly, there is a clear need for more powerful approaches.

### Proposal of the TZMD approach

**Fig. 1:**
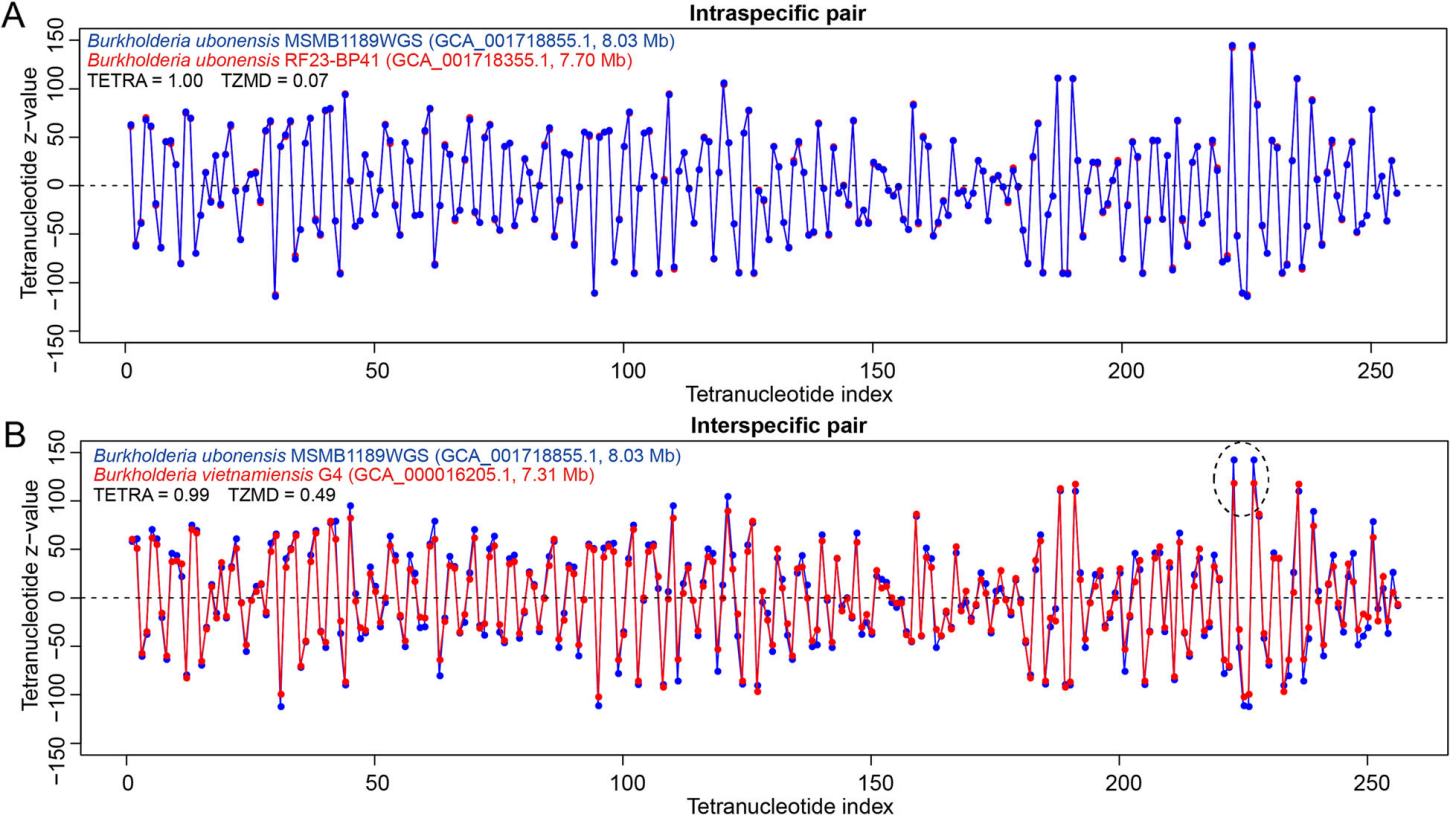
The TETRA approach cannot distinguish closely related species with only slightly different compositions. **a** For an interspecific pair. **b** For an intraspecific pair with different compositions. Boxed, distinct tetranucleotide-derived z-value difference

TETRA cannot differentiate closely related species, such as *Campylobacter jejuni* and *C.coli* [23], and most intraspecific strains, as shown by our above findings (Additional file 2: Figure S5). There are two possible reasons: one is that these organisms have almost no differences in composition and the other is that the resolution of TETRA is too low to distinguish these organisms, although they have different compositions. We found that some of the inability to differentiate closely related species or intraspecific strains was caused by the low resolution of TETRA. Taking the intraspecific pair *Burkholderia ubonensis* MSMB1189WGS and *B. ubonensis* RF23-BP41 and the interspecific pair *B. ubonensis* MSMB1189WGS and *B. vietnamiensis* G4 as examples, the intraspecific pair had almost identical composition yielding a TETRA value of 1.00, as the two curves almost completely coincided (Fig. 1a). Nevertheless, we found that TETRA also yielded an undistinguishable TETRA value of 0.99 for the interspecific pair with clearly different compositions (Fig. 1b), according to the above-determined cutoff of 0.99 (Additional file 2: Figure S1 and S3). This finding demonstrated that TETRA truly had low resolution to distinguish closely-related species, which was one of the limitations of the TETRA approach.

One possible reason for the low resolution of TETRA is that the Pearson correlation coefficient cannot efficiently measure the individual z-value difference, as demonstrated by the example shown in Fig. 1b delineated by a dotted oval. From a mathematical perspective, the Pearson correlation coefficient reflects a general trend for all 256 z-values, while the Manhattan distance efficiently reflects the z-value difference for each individual tetranucleotide (see Methods), implying that using the Manhattan distance instead of the Pearson correlation coefficient may improve the resolution to measure the composition difference. Accordingly, we proposed TZMD, a novel method using the Manhattan distance, and anticipated that it would increase the resolution for tetranucleotide usage biases.

**Fig. 2:**
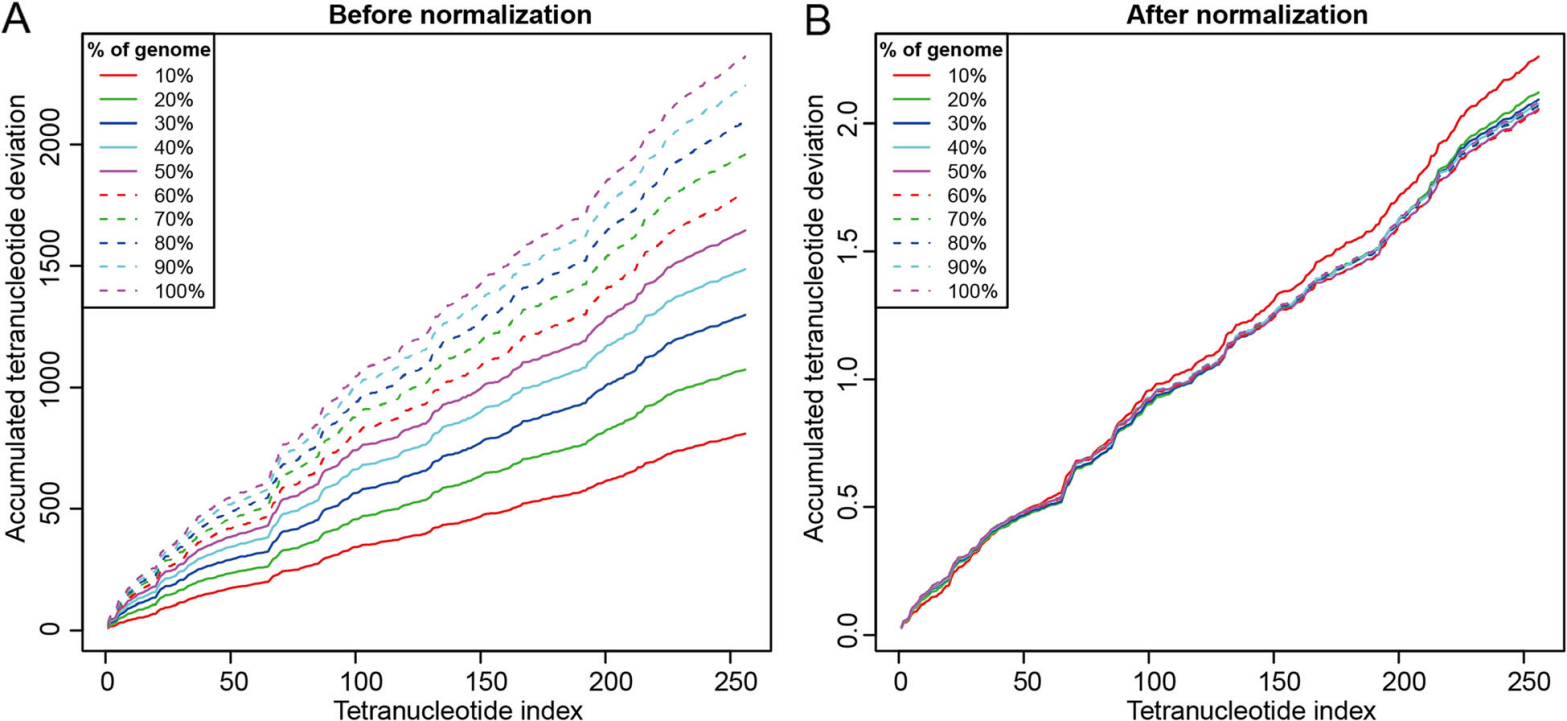
Normalization of tetranucleotide-derived z-values. **a** Before normalization. **b** After normalization. Values shown here represent the accumulated tetranucleotide deviations for *Buchnera aphidicola* str. APS (*Acyrthosiphon pisum*) (GCA_000009605.1)

When calculating z-values for 10-100% of the genome, we found that the tetranucleotide deviation (usage bias including over- and underrepresentation) increased with sequence size (Additional file 2: Figure S6A), which was more clearly demonstrated by using the accumulated tetranucleotide deviations (Fig. 2a). We showed that the sequence size greatly affected the TZMD (Additional file 1: Table S1), while it did not affect the TETRA. To eliminate the impact of sequence size, we normalized the z-values by dividing by the square root of the sequence size. After normalization, differently sized sequences from the same genome expectedly yielded similar deviations (Fig. 2b and Additional file 2: Figure S6B), although 10% of the genome generated relatively different deviations due to the skewed composition for short sequences (Fig. 2b). This finding demonstrated that our method for normalization is correct. Thus, the normalized z-values can be used for TZMD calculation since they accurately reflect genomic composition. We calculated the TZMD based on the normalized z-values of the aforementioned two pairs and found that our TZMD approach generated two distinguishable values (Fig. 1) according to the below-determined TZMD cutoff of 0.21 (see below), preliminarily showing that TZMD has a higher resolution than TETRA.

### Reflecting the maximal genomic difference

**Fig. 3:**
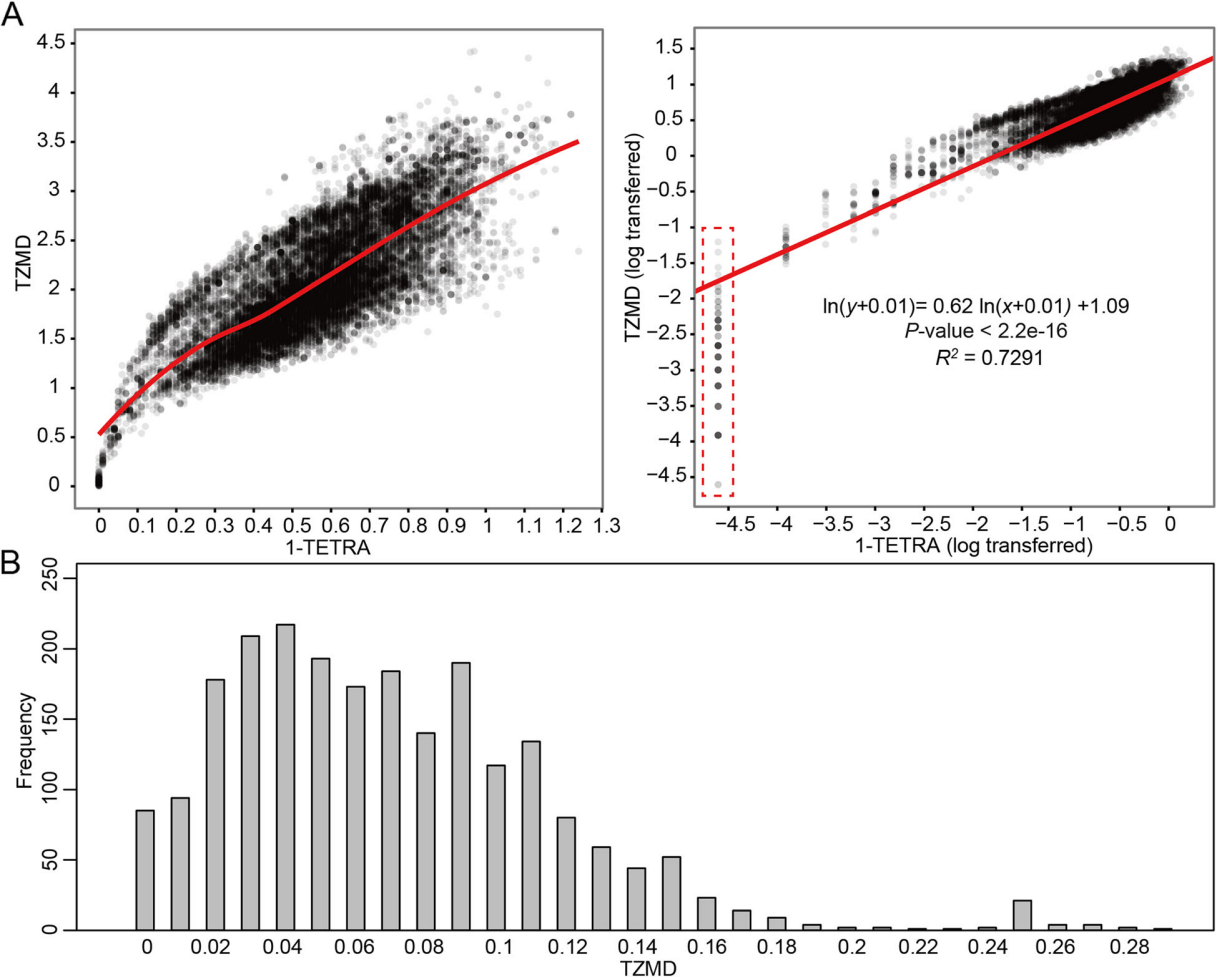
Relationship between TZMD and TETRA. **a** Correlation analysis between TZMD and TETRA. Left, without logarithmic transformation; right, after logarithmic transformation. All values were generated using 1779 queries and 264 references. **b** TZMD distribution for pairs with TETRA = 1, which are boxed by the broken line in Panel A

**Fig. 4:**
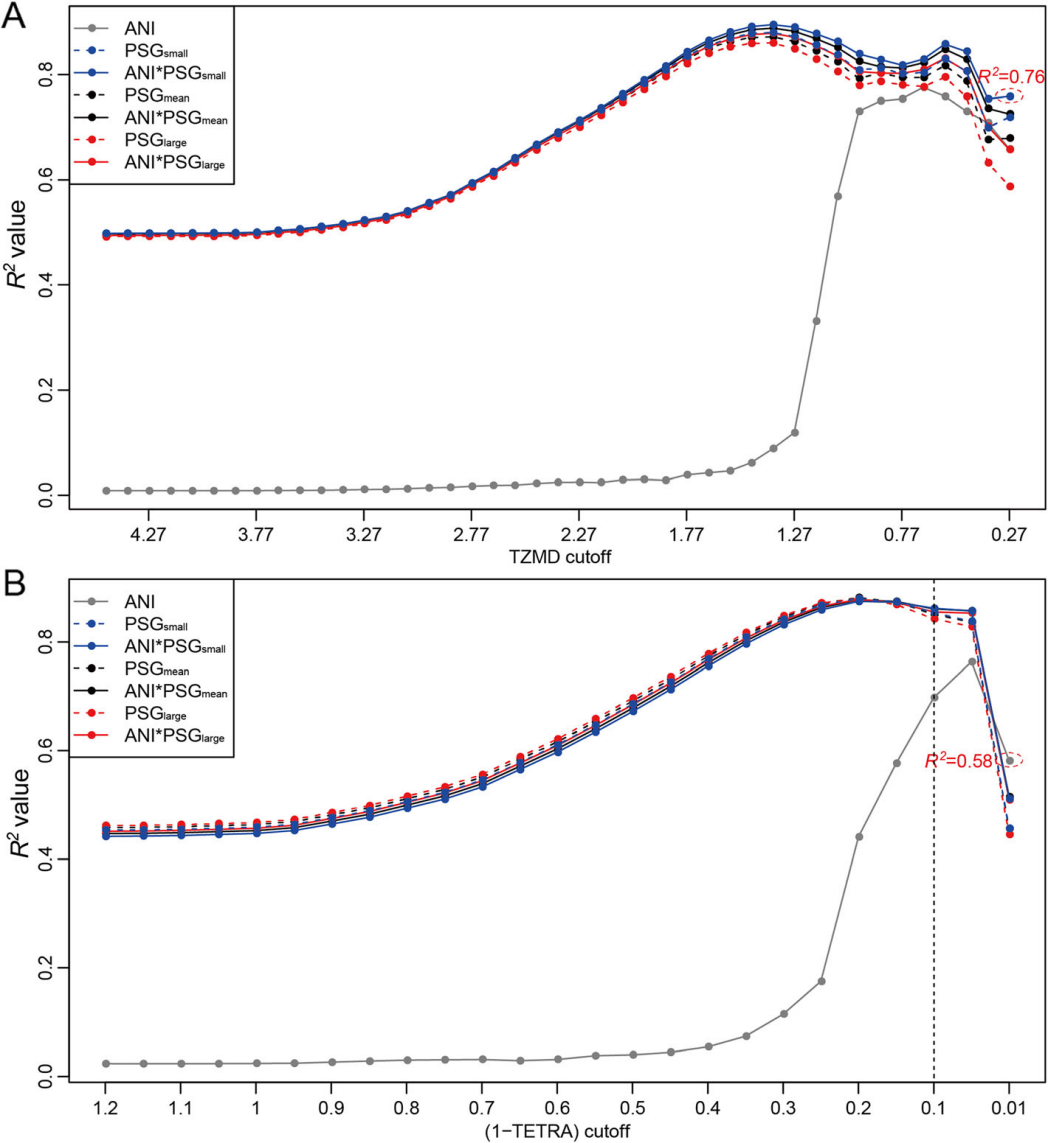
TZMD reflects the maximal genome difference. **a** Correlation results for TZMD. **b** Correlation results for TETRA. A total of 1779 queries against 264 references were used. The correlation results for organisms at or below the species level are indicated by the dotted ovals. TETRA gives the highest *R*^*2*^ values for measures other than the maximal difference (ANI*PSG_small_) except when TETRA > 0.90 (indicated by a vertical dotted line)

Although both TETRA and TZMD quantify composition similarity/difference, our results from the aforementioned 1779 queries against 264 references showed that TZMD was only moderately correlated with TETRA in a power fashion (*R*^*2*^ *=* 0.7291), indicating that TZMD is not simply a fine-tuned version of TETRA (Fig. 3a). Accordingly, we explored whether TZMD or TETRA was more robust in reflecting genomic differences. Genomic differences between two genomes encompass two aspects: the percentage of shared genome (PSG) and average nucleotide identity (ANI) of the shared genome. For a given pair, we calculated two PSGs: one for the smaller PSG (termed PSG_small_) and the other for the larger PSG (termed PSG_large_). In addition, we calculated a medium PSG that was an average of the two PSGs (termed PSG_mean_). In total, there were seven different measures including ANI, PSG_small_, PSG_mean_ and PSG_large_ for one aspect of genomic difference and ANI*PSG_small_, ANI*PSG_mean_ and ANI*PSG_large_ for two aspects of genomic difference. Among them, ANI*PSG_small_ was the maximal difference between two genomes. Our correlation analysis showed that TZMD showed the highest *R*^*2*^ values for the maximal genomic differences (ANI*PSG_small_), regardless of the TZMD cutoff used (Fig. 4a). In contrast, TETRA did not give the highest *R*^*2*^ values for ANI*PSG_small_ under almost all TETRA cutoffs except 0.1 (Fig. 4b). Thus, TZMD always reflected the maximal difference, endowing it with a higher distinguishing power than TETRA. Additionally, it was noteworthy that the *R*^*2*^ values for the maximal differences were only slightly higher than those for the other measures except the ANI for distantly related organisms, but relatively much higher for closely related organisms (Fig. 4a). This result indicates that the resolution difference between TZMD and TETRA arises primarily with closely related organisms, although TZMD also exhibits a slight improvement over TETRA for differentiating distantly related organisms.

Next, we explored whether TZMD was more robust in distinguishing similar organisms at or below the species level. Tracking for the 1779 queries against 264 references revealed that almost all intraspecific TETRA values were > 0.99 with only two exceptions: one for the *Borrelia hermsii* strains CC1 and HS1 with an atypical TETRA of 0.97 and the other for the *Borreliella burgdorferi* strains CA382 and B31 with an atypical TETRA of 0.95. Correspondingly, these exceptions had an atypical TZMD of 0.31 and 0.42 respectively (Additional file 2: Figure S7). Excluding these two pairs, all other intraspecific pairs had a maximal TZMD of 0.27, which was used as the TZMD cutoff to evaluate the distinguishing ability of TZMD for similar organisms. We found that TZMD yielded a higher *R*^*2*^ value under its cutoff of 0.27 than TETRA when its cutoff was set at 0.99, theoretically demonstrating that TZMD is more robust in distinguishing organisms at or below the species level, including species belonging to a single genospecies at the species level, and subspecies or intraspecific strains below the species level.

In conclusion, from the theoretical viewpoint, we demonstrated that TZMD has a higher resolution than TETRA. As an example, the pairs with a TETRA of 1.00, which were considered to have completely identical compositions by the TETRA approach, were found to show distinguishable TZMD values ranging from 0 to 0.29 (Fig. 3b), supporting that TZMD has a higher resolution than TETRA.

### Slight improvement in species differentiation

Genomic composition is species specific, and intraspecific differences are generally lower than interspecific differences [2, 9, 47, 48]. Thus, TETRA has been widely applied at or above the species level, such as in metagenomic binning [5, 28] and species differentiation [24]. However, although TETRA performs very well in most conditions, it cannot distinguish certain closely related species, especially intra-genus species [23]. One possible reason is that TETRA incompletely quantifies genomic differences (Fig. 4b). In contrast, TZMD reflects the maximal difference between genomes. Thus, we investigated whether TZMD could improve species differentiation.

**Fig. 5:**
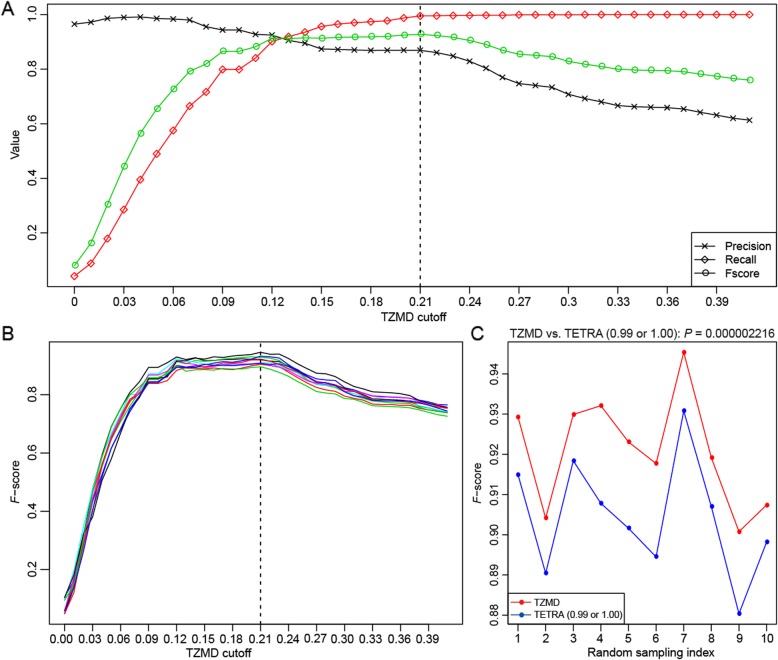
A comparison based on the *F*-score showed that TZMD slightly improved species differentiation. **a** Determining the TZMD cutoff with the highest *F*-score. **b** *F*-scores for 10 samplings with the TZMD method. **c** The highest *F*-scores for both TZMD and TETRA approaches. All 1779 queries against 264 references were used (Panel **a**). For each sampling, 200 intraspecific and 50,000 interspecific pairs were randomly sampled (Panels **b** and **c**). Because TETRA had two criteria (0.99 or 1.00) (Additional file 2: Figure S1A and S3A), the TETRA method using both criteria was compared. Dashed line, the TZMD cutoff at 0.21; *P*-value, one-tailed paired t-test

For species differentiation, we first determined the optimal species-level cutoff for TZMD. Here, we used the 1779 queries against 264 references to determine the optimal species-level cutoff. The optimal cutoff was determined to be 0.21 with both the highest *F*-score (precision = 0.8688, recall = 0.9949) (Fig. 5a) and the highest Rand index (Additional file 2: Figure S8A). Using the 0.21 criterion, TZMD correctly differentiated 1954 intraspecific and 467,397 interspecific pairs to achieve a high Rand index of ~ 0.9994, while TETRA correctly differentiated 1962 intraspecific and 466,776 interspecific or 1918 intraspecific and 467,371 interspecific pairs to achieve a relatively low Rand index of ~ 0.9980 or ~ 0.9992 (Additional file 2: Figure S8B) when using the 0.99 or 1.00 criterion respectively (Additional file 2: Figure S1A and S3A). For the statistical test, we randomly sampled 200 distinct intraspecific pairs and 50,000 distinct interspecific pairs for each sampling 10 times for both methods. The results showed that the optimal cutoff for TZMD could also be determined at 0.21 (Fig. 5b and Additional file 2: Figure S8C), which was in line with the above finding (Fig. 5a and Additional file 2: Figure S8A). A paired t-test showed that TZMD significantly outperformed TETRA (Fig. 5c and Additional file 2: Figure S8D). Therefore, from the perspective of species differentiation, we demonstrated that TZMD had a higher resolution than TETRA. However, compared with TETRA, TZMD exhibited only a slight improvement in species differentiation, possibly because the maximal genomic differences (ANI*PSG_small_) reflected by TZMD were only slightly superior to the PSG_large_ or other measures reflected by TETRA for distantly related organisms (Fig. 4a).

### Differentiating similar species belonging to a single genospecies

**Fig. 6:**
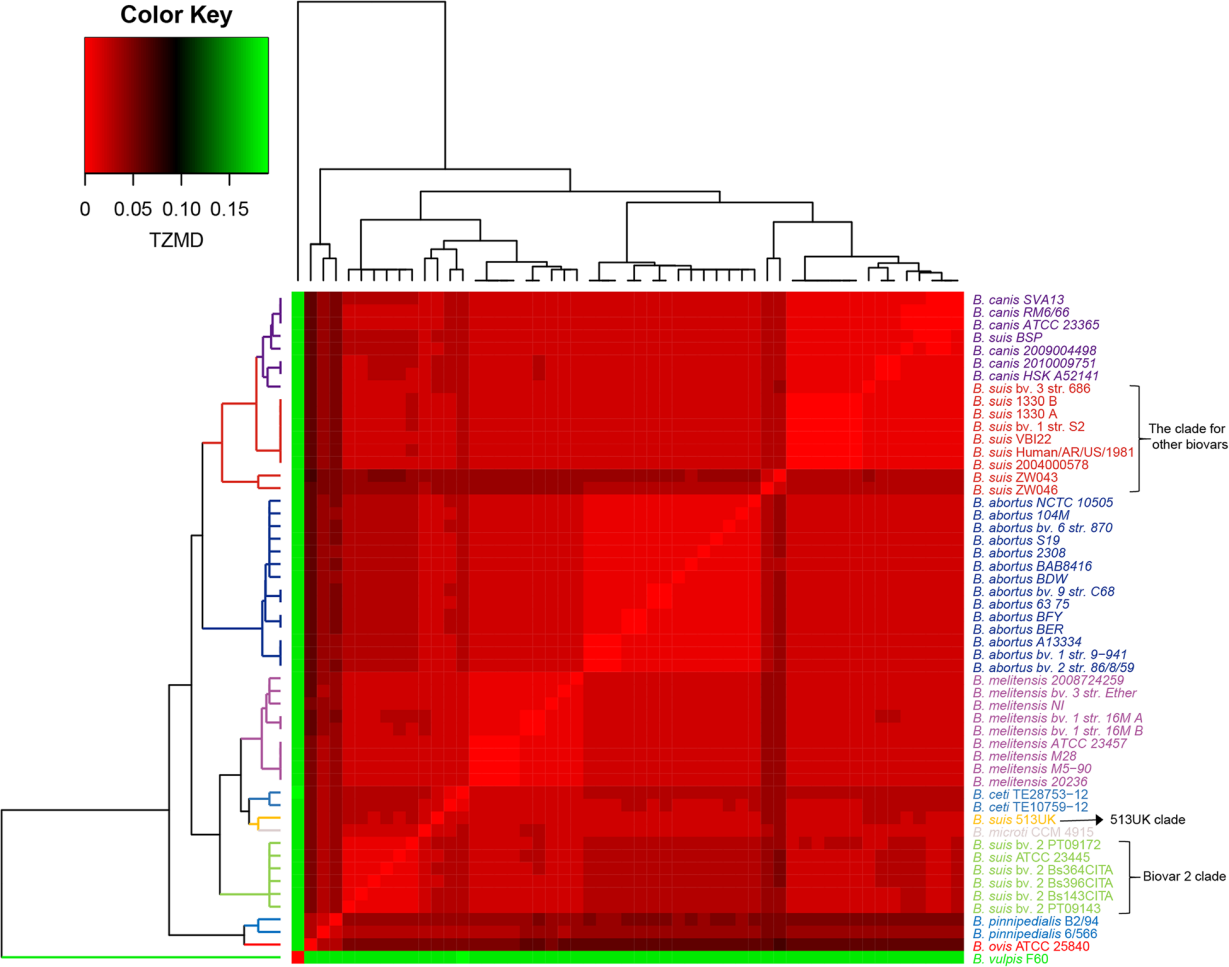
TZMD differentiates *Brucella* species. *Brucella* species are considered a single genospecies. The TZMD value is used as a basis for color intensity. Different colors for species names indicate different clades. Three main clades for *B. suis* are also indicated. The figure was drawn by using the heatmap.2 function (gplots package, ward.D2 linkage)

Closely related species, such as *Escherichia coli–Shigella* [24] and *Bacillus anthracis–Bacillus thuringiensis–Bacillus cereus* [46], were defined as a single genospecies by the methods based on overall genotypic similarity, such as the ANI approach. Generally, TETRA cannot distinguish most closely related species. As an alternative, we considered whether TZMD could differentiate between these species. *Brucella* species were taken as an example for testing, as they were delineated as a single species by the DNA-DNA hybridization method due to their > 90% DNA-DNA hybridization values [49, 50] and by the ANI approach due to their > 96% ANIs [46]. We collected 53 complete genomes from the National Center for Biotechnology Information (NCBI) database (Additional file 1: Table S3) and used them to test whether TZMD could differentiate similar species belonging to a single genospecies. As expected, TETRA could not differentiate any *Brucella* species (Additional file 2: Figure S9). However, strikingly, TZMD clearly differentiated all *Brucella* species (Fig. 6). In addition, TZMD further separated *B. suis* biovars into three main clades (513UK, biovar 2 and other biovars including biovars 1, 3 and 4), which was consistent with the phylogenetic results based on genome-wide single-nucleotide polymorphisms (SNPs), multilocus sequencing typing and whole-genome sequence alignment [51]. Additionally, TZMD showed that *B. canis* might have evolved from the clade of “other biovars” of *B. suis*, in line with the findings of a previous study [52]. In addition, the highest TZMD value was 0.19 for the pair *B. vulpis* F60 and *B. suis* bv. 1 str. S2, followed by 0.18 for the pairs of *B. vulpis* F60 with all other strains and < 0.1 for all other pairs (Fig. 6). Therefore, according to its optimal cutoff for species differentiation (Fig. 5a), TZMD only delineated all *Brucella* species as a single genospecies, in accordance with previous studies [46, 49, 50].

*Yersinia pseudotuberculosis*–*Yersinia pestis* and *Burkholderia mallei–Burkholderia pseudomallei*, both of which were considered as a single genospecies by the ANI approach [46], were used as two other examples. In total, 45 complete genomes of *Y. pseudotuberculosis*–*Y. pestis* (Additional file 1: Table S4) and 67 complete genomes of *B. mallei–B. pseudomallei* (Additional file 1 Table S5) were collected from the NCBI database for analysis. TETRA could not distinguish between *Y. pestis* and *Y. pseudotuberculosis* (Additional file 2: Figure S10). By contrast, TZMD clearly differentiated them (Additional file 2: Figure S11). Similar results were also obtained for *B. mallei–B. pseudomallei* (Additional file 2: Figure S13 and S14). In conclusion, all these findings at the species level demonstrated that TZMD has a higher resolution than TETRA.

### Differentiating subspecies and intraspecific strains

**Fig. 7:**
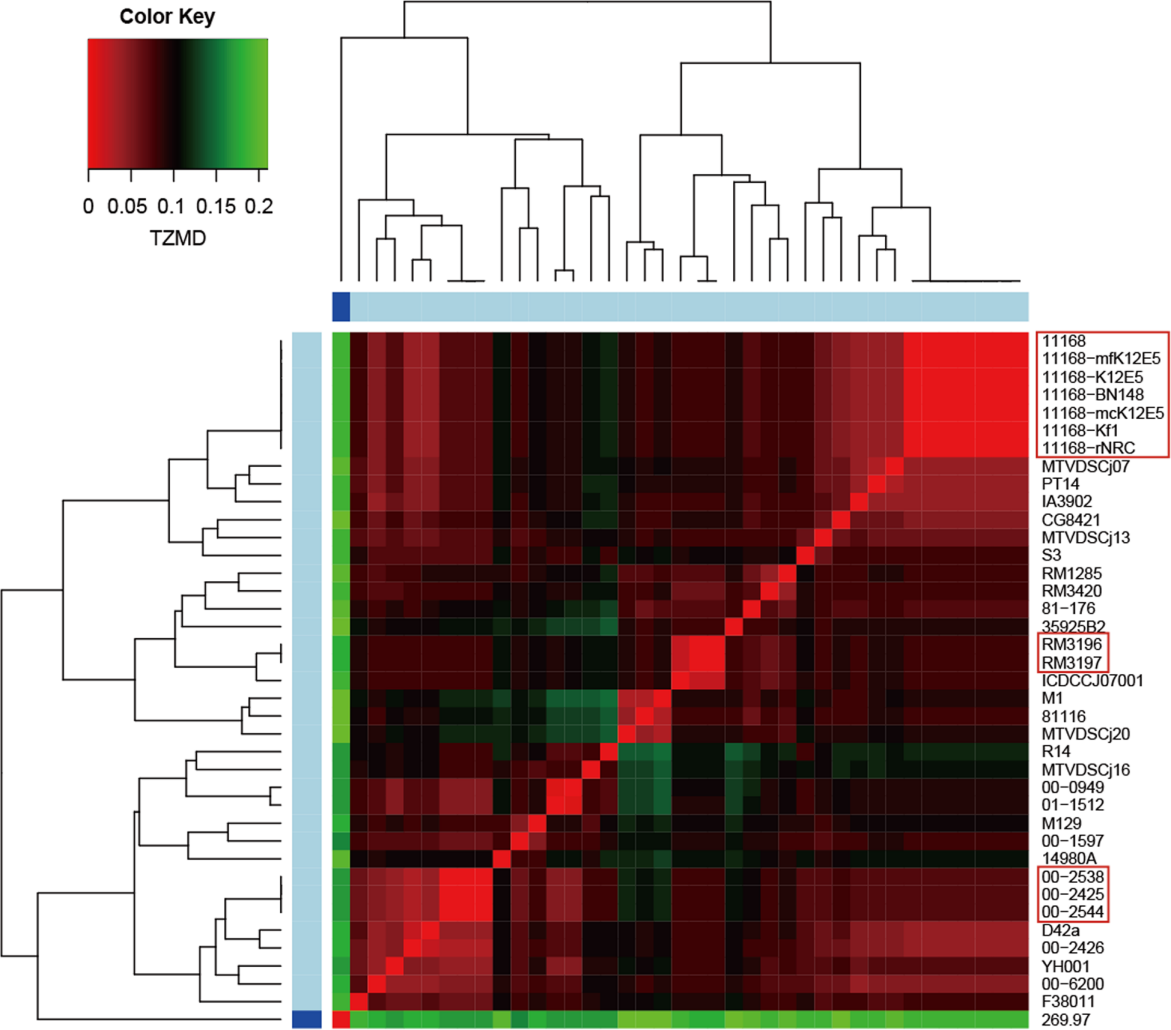
TZMD differentiates subspecies and intraspecific strains of *Campylobacter jejuni*. The TZMD value is used as a basis for color intensity. The boxed, clonal strains; blue bar, subsp. *doylei*; light blue bar, subsp. *jejuni*. The figure was drawn by using the heatmap.2 function (gplots package, ward.D2 linkage)

Next, we tested whether TZMD could be used to differentiate subspecies and intraspecific strains. *C. jejuni*, one of the major foodborne pathogens in the world, causes enteritis or Guillain–Barre syndrome in humans [53–55]. *C. jejuni* has two distinct subspecies: subsp. *jejuni* and subsp. *doylei* [56]. More importantly, both subspecies have been completely sequenced. In addition, the complete genomes of numerous strains of *C. jejuni* have been determined [23]. Therefore, *C. jejuni* was selected to explore this issue. We collected all 39 *C. jejuni* complete genomes from the NCBI database (Additional file 1: Table S6), one for subsp. *doylei* and the remaining for subsp. *jejuni*. Our results showed that the TZMD approach clearly distinguished the two subspecies as well as intraspecific strains (Fig. 7), while the TETRA approach could not differentiate any strain or even subspecies (Additional file 2: Figure S14).

*Francisella tularensis* has several subspecies, including subsp. *novicida*, subsp. *holarctica* and subsp. *tularensis*, which were selected to further compare the abilities of TZMD and TETRA to distinguish subspecies. In total, 33 complete genomes were collected (Additional file 1: Table S7). TZMD clearly differentiated all subspecies (Additional file 2: Figure S15), while TETRA could not differentiate subsp. *holarctica* and subsp. *novicida* (Additional file 2: Figure S16).

Additional cases, including 51 complete genomes of *Streptococcus pyogenes* (Additional file 1: Table S8) and 53 complete genomes of *Bacillus cereus* (Additional file 1: Table S9), were used to further compare the abilities of TZMD and TETRA to distinguish intraspecific strains. TZMD clearly distinguished intraspecific strains of *S. pyogenes* (Additional file 2: Figure S17), while TETRA could not differentiate any strain of *S. pyogenes* (Additional file 2: Figure S18). Similar results were also found for *B. cereus* (Additional file 2: Figure S19 and S20).

**Fig. 8:**
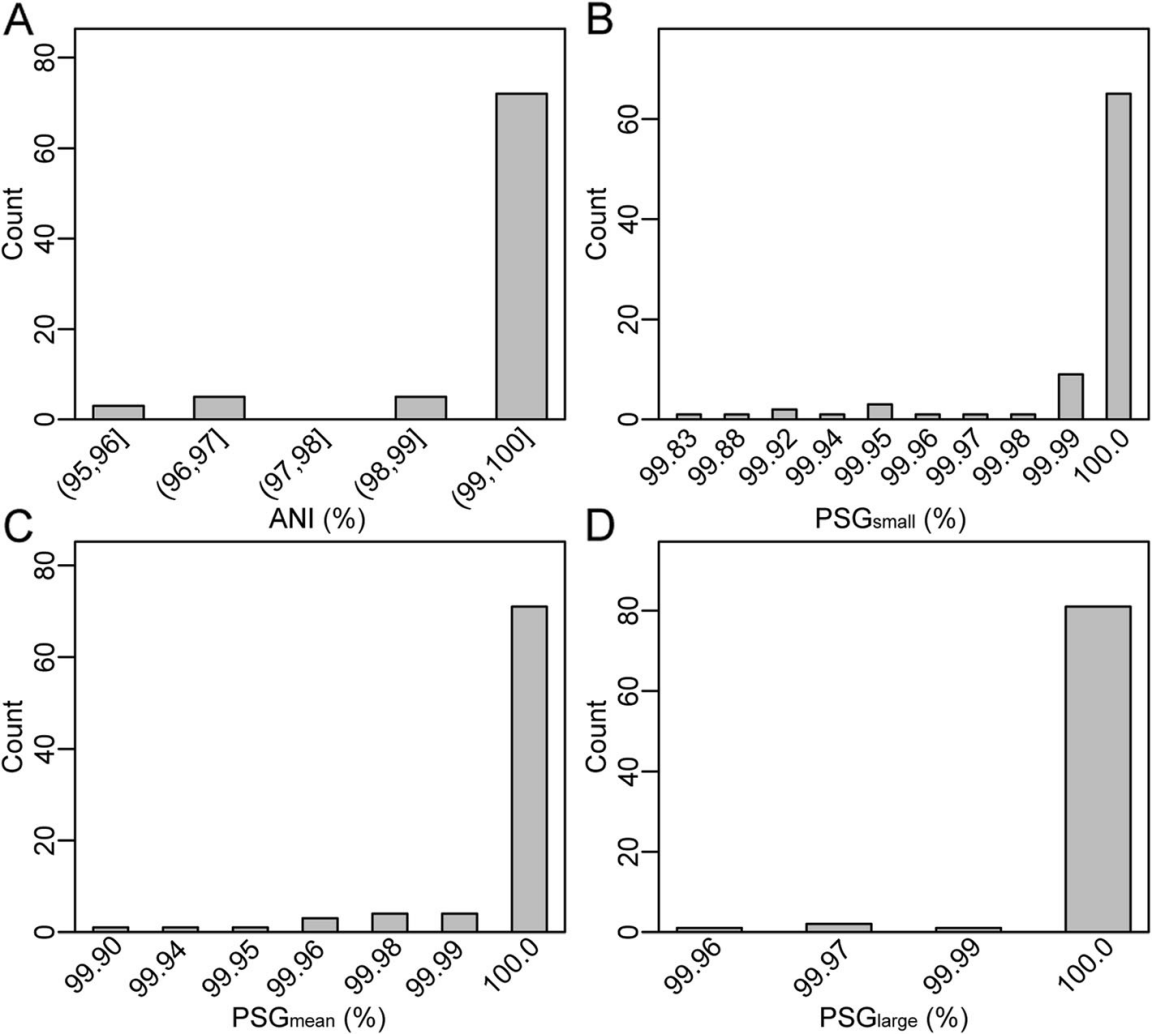
TZMD = 0 additionally includes high ANI and high PSG. **a**) ANI distribution. **b** PSG_small_ distribution. **c** PSG_mean_ distribution. **d** PSG_large_ distribution. All values shown here are summarized from pairs with TZMD = 0 from the 1779 queries against 264 references

In addition, we found that the TZMD approach further distinguished clonal and non-clonal strains for clonal strains with a TZMD value of 0 and non-clonal strains with TZMD > 0 (Fig. 7 and Additional file 2: Figure S17 and S19). Summarizing all pairs with a TZMD value of 0 from the 1779 queries against 264 references showed that this standard included substantially more information. This standard intrinsically ensured that clonal strains had considerably high ANIs (95.85-100%) (Fig. 8a), as well as considerably high PSGs (almost 100%, regardless of the PSG metric used) (Fig. 8b, c and d). Therefore, this standard substantially ensured that at least three aspects (composition, ANI and PSG) were extremely similar, showing that TZMD is powerful for detecting clonal strains with high confidence. All findings at the subspecies and strain levels demonstrated that TZMD has a higher resolution than TETRA.

## Discussion

### Demonstration of high resolution

In this study, we present TZMD, a high-resolution method for distinguishing genomic composition. We determined that TETRA was able to represent all other published statistical methods to profile tetranucleotide usage biases. Therefore, we only needed to compare our TZMD method with TETRA. We demonstrated the high resolution of TZMD by comparing it with the TETRA method from two viewpoints. First, from the theoretical point of view, we principally demonstrated that TZMD reflected the maximal difference between two genomes, endowing it with high resolution. Second, from the practical point of view, we assessed its high resolution for real data from four aspects, including species differentiation at or above the species level, differentiation of similar species belonging to a single genospecies at species level, subspecies below the species level and intraspecific strains below the species level. TZMD exhibited a slight improvement in species differentiation, possibly because the maximal genome differences reflected by TZMD are only slightly superior to the PSG_large_ or other measures reflected by TETRA for distantly related organisms (Fig. 4a). However, maximal genome differences reflected by TZMD are considerably superior to the PSG_large_ or other measures reflected by TETRA for closely-related organisms (Fig. 4a), which is why TZMD can differentiate species belonging to a single genospecies, subspecies and intraspecific strains, whereas TETRA is almost unable to differentiate them. Taken together, these findings demonstrated that TZMD has a high resolution.

### Strain-specific feature of composition

The studies for genomic composition began in the early 1960s [6]. Since then, almost all related studies have focused on the development of statistical methods and extensive demonstration and wide application of the species-specific nature of composition. In addition to the species-specific nature of composition [2, 9, 14, 19–21], our results showed that composition is also strain specific (Fig. 7). To our knowledge, this is the first study to demonstrate that genomic composition is strain specific. The strain-specific nature of composition is possibly due to (but not limited to) various factors. First, bacteria are evolving, which may result in SNPs, insertions or deletions. Second, some genes are acquired through horizontal gene transfer [57]. For example, genomic islands, which are horizontally transferred, carry biased composition from their core genomes [58]. Third, some genes are lost through genomic reduction [59]. Fourth, some plasmids are conjugative [60, 61], which may cause varied composition for their recipient genomes [41].

### Strain typing

Almost all previously developed methods can distinguish only genomic composition at or above the species level, leaving a long-standing need to develop methods capable of strain-level differentiation. As far as we know, TZMD is the first genomic composition-based algorithm with the ability to differentiate similar species, subspecies and intraspecific strains and thus can be used for strain typing. Similar species, in a sense, are special intraspecific strains belonging to a single genospecies, as their TZMD values are < 0.21 (Fig. 6). Composition Vector Tree (CVTree) can also distinguish intraspecific strains for strain typing [62]. However, CVTree uses the proteomic composition (oligopeptide) rather than the genomic composition (oligonucleotide). Accordingly, TZMD is more convenient than CVTree, as TZMD directly uses genomes, while CVTree uses proteomes requiring prediction and translation of protein-coding genes from their genomes. In addition, CVTree requires 12,500 times the computational burden of TZMD, as the composition vector of dimension for CVTree is 20^5^ [62], while that for TZMD is only 4^4^. However, our results from the present study show that TZMD is sufficiently robust to distinguish closely related species, subspecies or strains without increasing the computational burden.

Furthermore, TZMD is a powerful standard to distinguish clonal and non-clonal strains. Traditional methods are mainly based on SNPs. For example, multilocus sequence typing uses SNPs from several housekeeping genes [63]. Compared with these traditional SNP-based methods, TZMD has at least four main advantages. First, SNP-based methods only consider the information of nucleotide identity, while the TZMD standard considers at least three aspects, including ANI, PSG and composition. Second, the TZMD standard is based on genome-wide information, outperforming traditional methods based only on single or several housekeeping genes. Third, TZMD is an easy-to-use method because its standard is set at 0, while it is difficult to set the exact SNP cutoffs for strain typing for traditional SNP-based methods. For methods using genome-wide information, the SNP cutoffs are challenging to set as the SNP cutoffs are species specific, requiring large-scale sequencing to determine the cutoffs for each species; for methods using single or multiple genes, the SNP cutoffs are also challenging to set even for the same species, as the thresholds may be different for different genes because they may be under different evolutionary stresses. Fourth, TZMD is an alignment-free method that may run faster than some traditional methods, especially genome-wide approaches, and thus reduces computing cost [24]. Taken together, these factors showed that TZMD is a powerful tool and the first easy-to-use approach for differentiating clonal and non-clonal strains.

### Impact of genome incompleteness

Incomplete genomes contain only part of their genomes and thus may carry a skewed composition to yield different TZMD and TETRA values related to their full genomes (Additional file 1: Table S1). Even genomes with 90% completeness may yield slightly different TZMD values (Additional file 1: Table S1), whereas most closely related organisms, including similar species belonging to a single genospecies, subspecies and intra-species strains, have only slightly different compositions (Figs. 6 and 7). Thus, using incomplete genomes may result in incorrect conclusions for differentiating these closely related organisms, which is why we only used complete genomes in this study. Therefore, the differentiation of closely related organisms requires complete genomes.

In contrast, genomic incompleteness has less of an impact on species differentiation. Nevertheless, genomic incompleteness may still affect the results. Although species differentiation using the < 0.21 criterion for TZMD and the > 0.99 or 1.00 criterion for TETRA is tolerant of some incompleteness, it is still affected by genomic completeness (Additional file 2: Figure S21). However, TZMD is more susceptible to genome incompleteness than TETRA. Thus, TZMD may be inferior to TETRA when analysing incomplete genomes, although TZMD slightly improves species differentiation when all tested genomes are complete (Fig. 5). However, both TZMD and TETRA are affected by genomic incompleteness, requiring the development of novel approaches.

### Metagenomic binning

Composition has been widely used for metagenomic binning [25–31], as it was species specific [2, 9, 19–21]. TETRA has been used for binning [5, 28, 64], and the Euclidean distance has also been used for binning [26, 65]. As the Manhattan distance has been reported to outperform the Euclidean distance [66], we herein only compared TZMD with TETRA. Our testing showed that TZMD performed worse than TETRA when fragment size was < 80 kb (Additional file 2: Figure S22), possibly because TZMD is more greatly affected by genome incompleteness than TETRA (Additional file 2: Figure S21), but it outperformed TETRA when fragment size was > 80 kb, possibly because TZMD performs slightly better in species differentiation. However, the difference of binning performance between them is very slight. Thus, either approach can be selected for binning.

## Conclusions

Here, we develop a novel approach termed TZMD for genomic composition and extensively demonstrate that TZMD is a high-resolution method. Although traditional approaches such as TETRA are effective in distinguishing most species and thus can be successfully applied for species differentiation, TZMD slightly improves these applications. Most importantly, TZMD exclusively extends the application of composition from the species level to the strain level, endowing it with the ability to differentiate species belonging to a single species, subspecies and intraspecific strains. Furthermore, TZMD is a powerful tool and the first easy-to-use approach for differentiating clonal and non-clonal strains. Additionally, this study is the first to show that composition is strain specific. Therefore, we hope that TZMD will be used alone or in combination with TETRA to facilitate bacterial studies in the future.

## Methods

### Calculation of PSG and ANI

Genomes were aligned using the NUCmer tool (version 3.23) [67], with default parameters except for “--maxmatch”. Then, we used the “.delta” file to calculate PSG and ANI. Stretches, such as paralogs and other repeats, overlapped in the alignment, which would introduce biases in PSG. Thus, the PSG for either genome of a pair was calculated as follows:

$$ PSG=\frac{\sum Aligned\ positions}{L} $$

where the numerator is the summed aligned positions (in terms of base pairs) and the denominator is the genomic size (also in terms of base pairs). Thus, we obtained two PSGs for a given pair: the smaller one termed PSG_small_ and the larger one termed PSG_large_. Additionally, an average PSG was devised and calculated as follows:

$$ {PSG}_{mean}=\frac{AP1+ AP2}{L1+L2} $$

where *AP1* and *L1* are the summed aligned positions and genomic size for the first genome respectively, and *AP2* and *L2* are for the second genome.

### Genome selection

All prokaryotic genome sequences listed at http://www.ncbi.nlm.nih.gov/genome/ were downloaded (on 20 January 2017). Then, we selected complete genomes with only one chromosome. After this step, 5819 genomes remained. To determine the TZMD cutoff at the species level, we selected 2043 genomes with unambiguous species relationships (> 96% ANI [24] and > 70% of the shared gene content [68]). These genomes were separated into 1779 query genomes (Additional file 1: Table S1) and 264 reference genomes (Additional file 1: Table S2), comprising 1964 intra- and 467,692 inter-species pairs.

### Statistics of observed oligonucleotide frequencies

In all cases, due to strand compositional asymmetry in certain bacterial genomes [69], all tested genomes were concatenated with their inverted complements and then processed through discarding ambiguous nucleotides (that is, not A, T, C, or G). The resulting sequences were compiled by moving a single base per step in the 5′ to 3′ direction with a length of *n* nucleotides (*n* is 4, 3, 2 or 1).

### Four statistical methods for tetranucleotide usage biases

We denote the observed frequency of an oligonucleotide as F*(.) (* indicates the oligonucleotide frequency computed from the genome extended with its reverse complementary sequence). For example, the observed frequency of the tetranucleotide XYZW and its component mononucleotide X were denoted as F*(XYZW) and F*(X) respectively. Then, the tetranucleotide usage biases assessed by the zero-order Markov method were written as follows [42]:

$$ \frac{F\ast (XYZW)}{F\ast (X)F\ast (Y)F\ast (Z)F\ast (W)} $$

The method of maximal-order Markov model can be calculated similarly with the following formula [42]:

$$ \frac{F\ast \left( XYZ W\right)F\ast (YZ)}{F\ast (XYZ)F\ast \left( YZ W\right)} $$

The method of relative tetranucleotide frequency can be calculated as follows [20]:

$$ \frac{F\ast \left( XY Z W\right)F\ast (XY)F\ast (XNZ)F\ast \left({XN}_1{N}_2W\right)F\ast (YZ)F\ast (YNW)F\ast (ZW)}{F\ast \left( XY Z\right)F\ast \left( XY NW\right)F\ast \left( XNZ W\right)F\ast \left( YZ W\right)F\ast (X)F\ast (Y)F\ast (Z)F\ast (W)} $$

where *N* is any nucleotide.

The tetranucleotide usage biases can also be measured as z-value Z*(XYZW) by the z-value method and can be calculated as follows [5]:

$$ Z\ast (XYZW)=\frac{F\ast (XYZW)-E\ast (XYZW)}{\sqrt{\operatorname{var}\left(F\ast (XYZW)\right)}} $$

where E*(XYZW) can be calculated using a maximal-order Markov model as follows [70]:

$$ E\ast \left( XYZ W\right)=\frac{F\ast (XYZ)F\ast \left( YZ W\right)}{F\ast (YZ)} $$

var(F*(XYZW) can be calculated as follows [5]:

$$ \operatorname{var}\left(F\ast \left( XYZ W\right)\right)=\frac{E\ast \left( XYZ W\right)\left[F\ast (YZ)-F\ast (XYZ)\right]\left[F\ast (YZ)-F\ast \left( YZ W\right)\right]}{F\ast {(YZ)}^2} $$

Then, tetranucleotide usage biases calculated from each statistical method were subjected to calculation of the Pearson correlation coefficient to measure the composition similarity between two genomes.

### TZMD calculation

The resulting sequences were subjected to calculation of tetranucleotide-derived z-values and TETRA values according to the method published by Teeling et al. [5]. If we denote the z-value of the *k*th tetranucleotide from the sequence *s* as *Z*_*s,k*_ and the corresponding normalized z-value as *NZ*_*s,k*_, then the z-value can be normalized as follows:

$$ {NZ}_{s,k}={Z}_{s,k}/\sqrt{l} $$

where *l* is the total length of the sequence extended with its reverse complement.

Subsequently, *TZMD* was calculated to measure the compositional difference between two sequences *s*_*1*_ and *s*_*2*_ as follows:

$$ TZMD=\sum \limits_{k=1}^{256}\left|{NZ}_{s_1,k}-{NZ}_{s_2,k}\right| $$

### Determination of the optimal cutoffs for species differentiation

Precision-recall and *F*-score were applied successfully to determine the optimal sequence similarity thresholds for 40 single-copy phylogenetic marker genes [45] and 16S rRNA genes [46] for species delineation of prokaryotes. Similarly, we used this strategy to determine the optimal TZMD cutoff for species differentiation. Briefly, all pairwise TZMD values were assigned into four categories given a threshold of *Y* TZMD (0-0.42 at 0.01 intervals): true positives (TP) for intraspecific pairs (> 96% ANI [24] and > 70% of the shared gene content [68]) with TZMD <*Y*; false negatives (FN) for intraspecific pairs with TZMD >*Y*; false positives (FP) for interspecific pairs with TZMD <*Y* and true negatives (TN) for interspecific pairs with TZMD >*Y*. The optimal threshold was obtained by maximizing the sensitivity (recall) as well as the precision to achieve the highest *F-*score, which is a harmonic mean of precision and recall. The *F*-score was calculated as follows:

$$ F\hbox{-} \mathrm{score}=2\left(\mathrm{precision}\times \mathrm{recall}\right)/\left(\mathrm{precision}+\mathrm{recall}\right) $$

where precision and recall were calculated as follows:

$$ \mathrm{precision}=\mathrm{TP}/\left(\mathrm{TP}+\mathrm{FP}\right) $$

$$ \mathrm{recall}=\mathrm{TP}/\left(\mathrm{TP}+\mathrm{FN}\right) $$

In addition, the Rand index, which is simply the number of correct assigned pairs (TP + TN) divided by the total number of tested pairs (TP + FN + FP + TN), was used to select the optimal threshold with the highest Rand index.

The optimal species-level thresholds for the above four statistical methods were also determined with the highest *F*-scores and Rand indexes.

## Supplementary information

**Additional file 1: Table S1.** All 1,779 complete query genomes. **Table S2.** All 264 complete reference genomes. **Table S3.** A list of complete *Brucella* genomes used in this study. **Table S4. ** A list of *Y. pestis* and *Y. pseudotuberculosis* genomes used in this study. **Table S5.** A list of *B. mallei* and *B. pseudomallei* genomes used in this study. **Table S6.*** Campylobacter jejuni* genomes used in this study. **Table S7.*** Francisella tularensis* genomes used in this study. **Table S8.*** Streptococcus pyogenes* genomes used in this study. **Table S9.*** Bacillus cereus* genomes used in this study.

**Additional file 2: Figure S1.** Determination of the optimal species-level cutoffs for the four published methods based on *F*-score. **Figure S2.** *F*-scores for 10 samplings for each method. **Figure S3.** Determination of the species-level cutoffs for the four published methods based on Rand index. **Figure S4.** Rand indexes for 10 samplings for each method. **Figure S5.** Instraspecific Pearson correlation coefficient distribution for each method. **Figure S6.** Normalization of tetranucleotide-derived z-values. **Figure S7.** Histrogram showing the TZMD distributions for both intraspecific and interspecific pairs. **Figure S8.** Comparison base on Rand index showed TZMD slightly improved species differentiation. **Figure S9.** TETRA cannot differentiate *Brucella* species belonging to one single genospecies. **Figure S10.** TETRA cannot differentiate *Y. pseudotuberculosis* and *Y. pestis* belonging to one single genospecies. **Figure S11.** TZMD differentiates *Y. pestis* and *Y. pseudotuberculosis* belonging to one single genospecies. **Figure S12.** TETRA cannot differentiate *B. mallei* and *B. pseudomallei* belonging to one single genospecies. **Figure S13.** TZMD differentiates *B. mallei* and *B. pesudomallei belonging to one single genospecies*. **Figure S14.** TETRA cannot differentiates sub species and intraspecific strains of *Campylobacter jejuni*. **Figure S15.** TZMD differentiates all subspecies of *Franscisella tularensis*. **Figure S16.** TETRA cannot differentiate two subspecies of *Francisella tularensis*. **Figure S17.** TZMD distinguishes intraspecific strains of *Streptococcus pyogenes*. **Figure S18.** TETRA cannot differentiate intraspecific strains of *Streptococcus pyogenes.* **Figure S19.** TZMD distinguishes intraspecific strains of *Bacillus cereus.* **Figure S20.** TETRA cannot differentiate intraspecific strains of *Bacillus cereus*. **Figure S21.** Impact of genomic completeness on TZMD and TETRA for species differentiation. **Figure S22.** Binning performance of TZMD and TETRA. **Table S1.** TZMD and TETRA values for differently-sized genomes.

## Data Availability

All data generated or analyzed during this study are included supplementary information files (see tables in Additional file 1). Additionally, the TZMD approach and all tested data are available at https://github.com/Yizhuangzhou/TZMD.

## Abbreviations

ANI: Average nucleotide identity
CVTree: Composition Vector Tree
FN: False negatives
FP: False positives
NCBI: National Center for Biotechnology Information
PSG: Percentage of shared genome
PSG_large_: The larger PSG
PSG_mean_: Average of the two PSGs
PSG_small_: The smaller PSG
SNP: Single-nucleotide polymorphism
TETRA: Tetranucleotide-derived z-value Pearson correlation coefficient
TN: True negatives
TP: True positives
TZMD: Tetranucleotide-derived Z-value Manhattan Distance
