# Supplementary material for: A high-resolution genomic composition-based method with the ability to distinguish similar bacterial organisms
Source: BMC Genomics. 2019 Oct 21;20:754. doi: 10.1186/s12864-019-6119-x (PMC6805505; doi:10.1186/s12864-019-6119-x)
Supplement: Supplementary file 2 — Additional file 2: Figure S1. Determination of the optimal species-level cutoffs for the four published methods based on F-score. Figure S2. F-scores for 10 samplings for each method. Figure S3. Determination of the species-level cutoffs for the four published methods based on Rand index. Figure S4. Rand indexes for 10 samplings for each method. Figure S5. Instraspecific Pearson correlation coefficient distribution for each method. Figure S6. Normalization of tetranucleotide-derived z-values. Figure S7. Histrogram showing the TZMD distributions for both intraspecific and interspecific pairs. Figure S8. Comparison base on Rand index showed TZMD slightly improved species differentiation. Figure S9. TETRA cannot differentiate Brucella species belonging to one single genospecies. Figure S10. TETRA cannot differentiate Y. pseudotuberculosis and Y. pestis belonging to one single genospecies. Figure S11. TZMD differentiates Y. pestis and Y. pseudotuberculosis belonging to one single genospecies. Figure S12. TETRA cannot differentiate B. mallei and B. pseudomallei belonging to one single genospecies. Figure S13. TZMD differentiates B. mallei and B. pesudomallei belonging to one single genospecies. Figure S14. TETRA cannot differentiates sub species and intraspecific strains of Campylobacter jejuni. Figure S15. TZMD differentiates all subspecies of Franscisella tularensis. Figure S16. TETRA cannot differentiate two subspecies of Francisella tularensis. Figure S17. TZMD distinguishes intraspecific strains of Streptococcus pyogenes. Figure S18. TETRA cannot differentiate intraspecific strains of Streptococcus pyogenes. Figure S19. TZMD distinguishes intraspecific strains of Bacillus cereus. Figure S20. TETRA cannot differentiate intraspecific strains of Bacillus cereus. Figure S21. Impact of genomic completeness on TZMD and TETRA for species differentiation. Figure S22. Binning performance of TZMD and TETRA. Table S1. TZMD and TETRA values for differently-sized genomes. [file 12864_2019_6119_MOESM2_ESM.pdf]

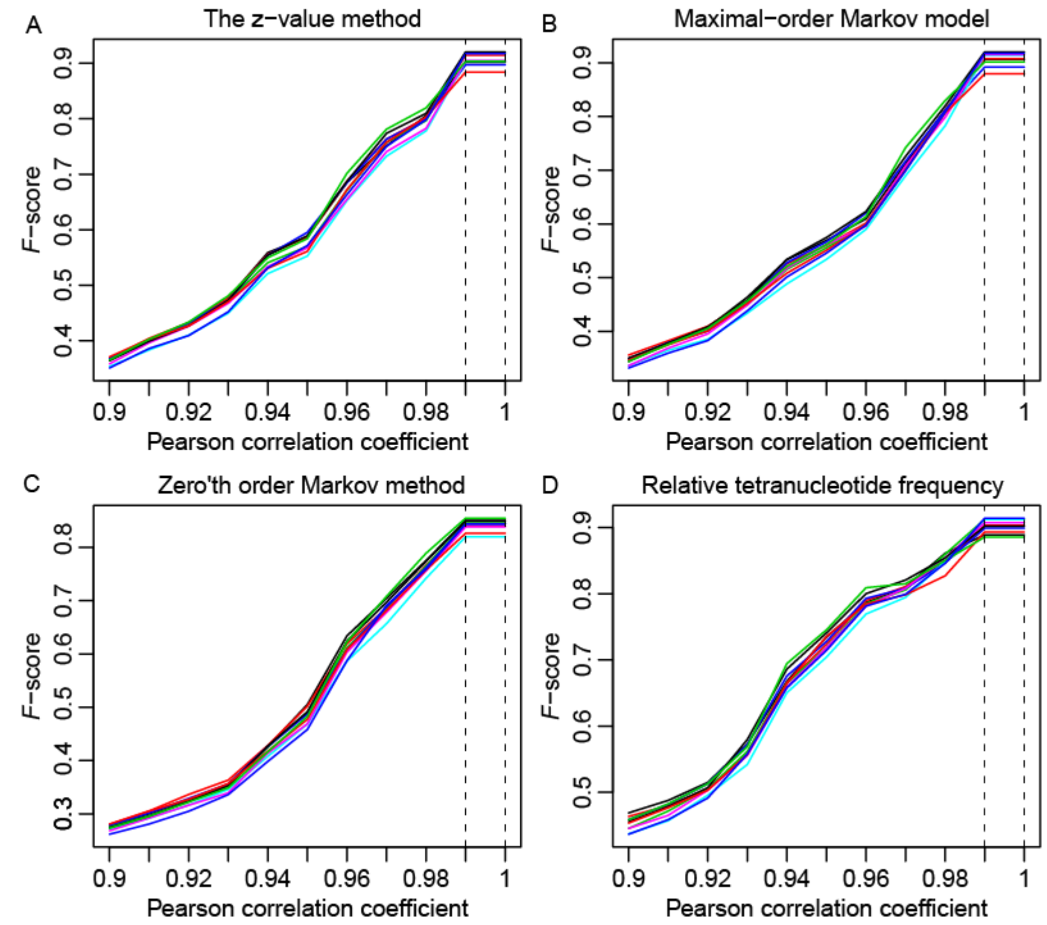

Figure S1. Determination of the optimal species-level cutoffs for the four published methods based on *F*-score. A, for the z-value method; B, for maximal-order Markov model; C, for zero-order Markov method; D, for relative tetranucleotide frequency. A total of 1,779 query genomes against 264 reference genomes, comprising 1,964 intraspecific pairs and 467,692 interspecific pairs, were used. Then, we randomly sampled 200 distinct intraspecific pairs and 50,000 distinct interspecific pairs for each sampling 10 times for each method. Vertical dashed line, the optimal species-level Pearson correlation coefficient cutoff.

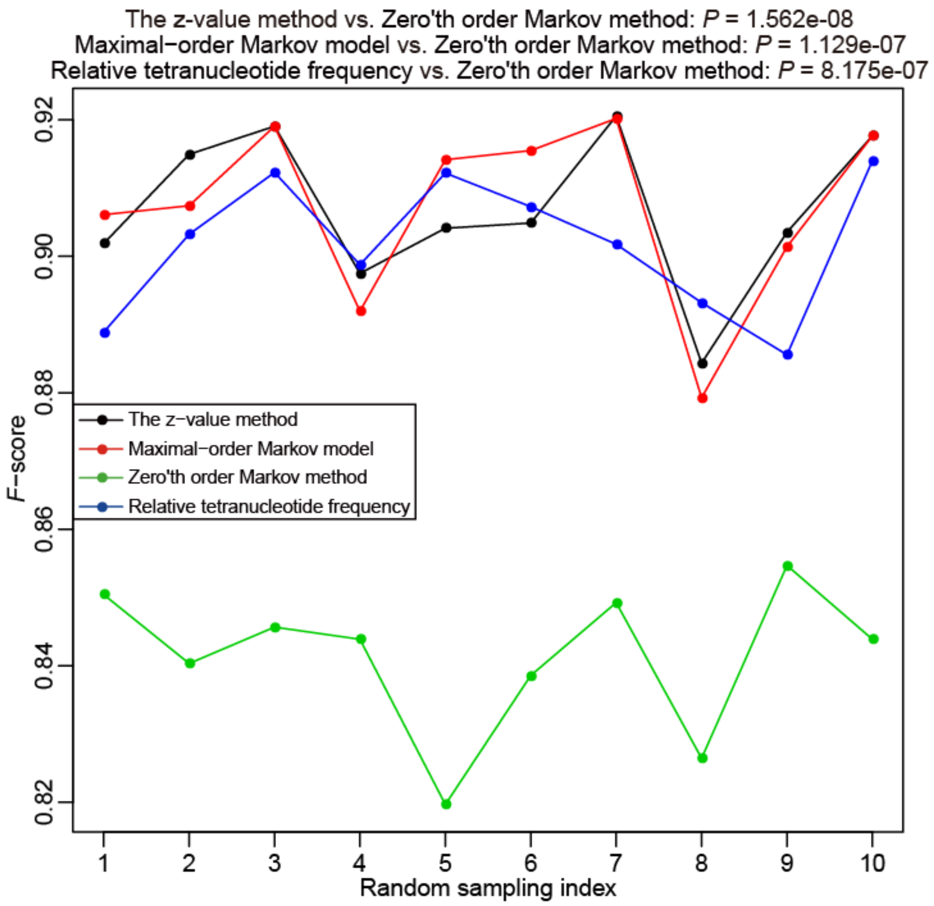

Figure S2. *F*-scores for 10 samplings for each method. A total of 1,779 query genomes against 264 reference genomes, comprising 1,964 intraspecific pairs and 467,692 interspecific pairs, were used. Then, we randomly sampled 200 distinct intraspecific pairs and 50,000 distinct interspecific pairs for each sampling 10 times for each method. *P*-values, one-tailed paired t-test.

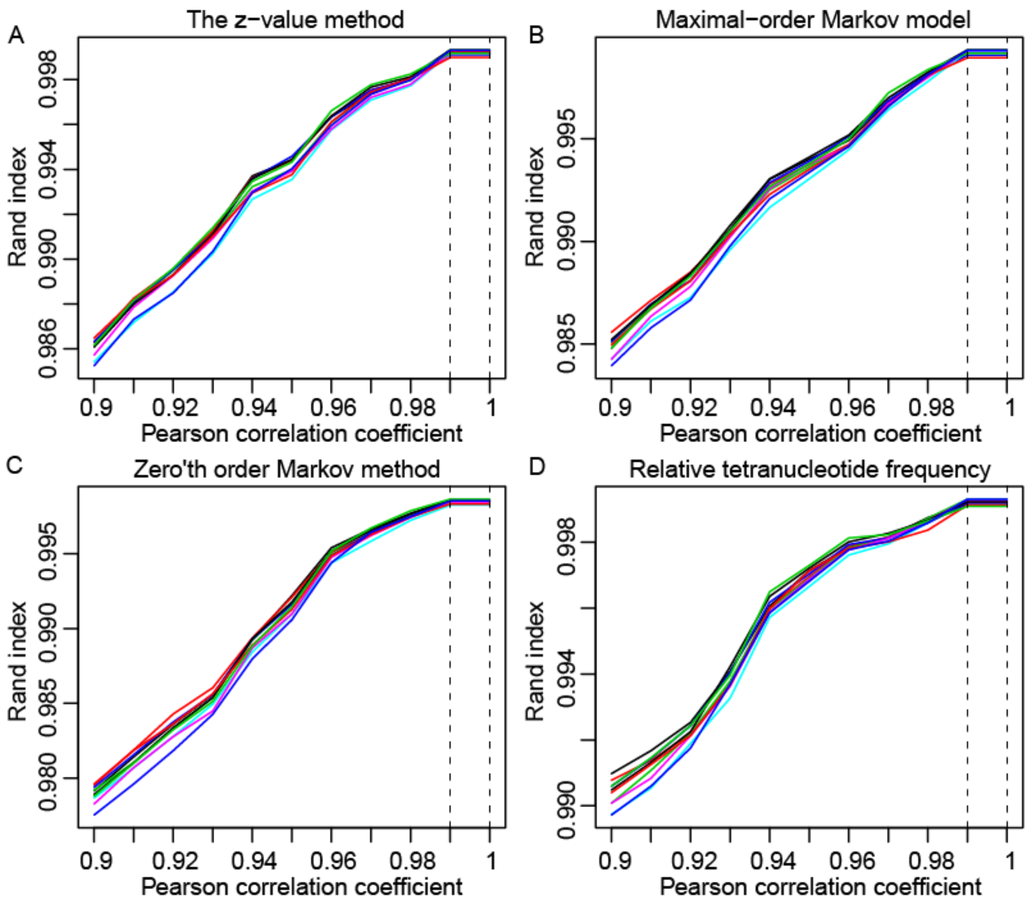

Figure S3. Determination of the species-level cutoffs for the four published methods based on Rand index. A, for the z-value method; B, for maximal-order Markov model; C, for zero-order Markov method; D, for relative tetranucleotide frequency. A total of 1,779 query genomes against 264 reference genomes, comprising 1,964 intraspecific pairs and 467,692 interspecific pairs, were used. Then, we randomly sampled 200 distinct intraspecific pairs and 50,000 distinct interspecific pairs for each sampling 10 times for each method. Vertical dashed line, the optimal species-level Pearson correlation coefficient cutoff.

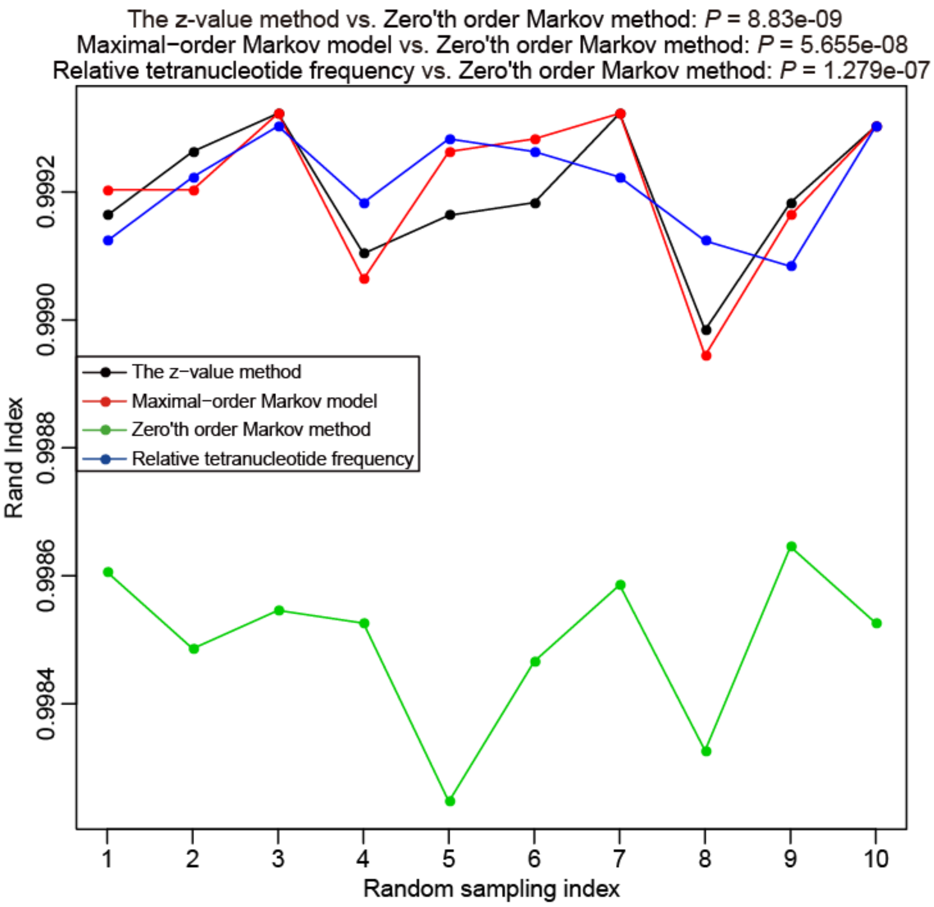

Figure S4. Rand indexes for 10 samplings for each method. A total of 1,779 query genomes against 264 reference genomes, comprising 1,964 intraspecific pairs and 467,692 interspecific pairs, were used. Then, we randomly sampled 200 distinct intraspecific pairs and 50,000 distinct interspecific pairs for each sampling 10 times for each method. *P*-values, one-tailed paired t-test.

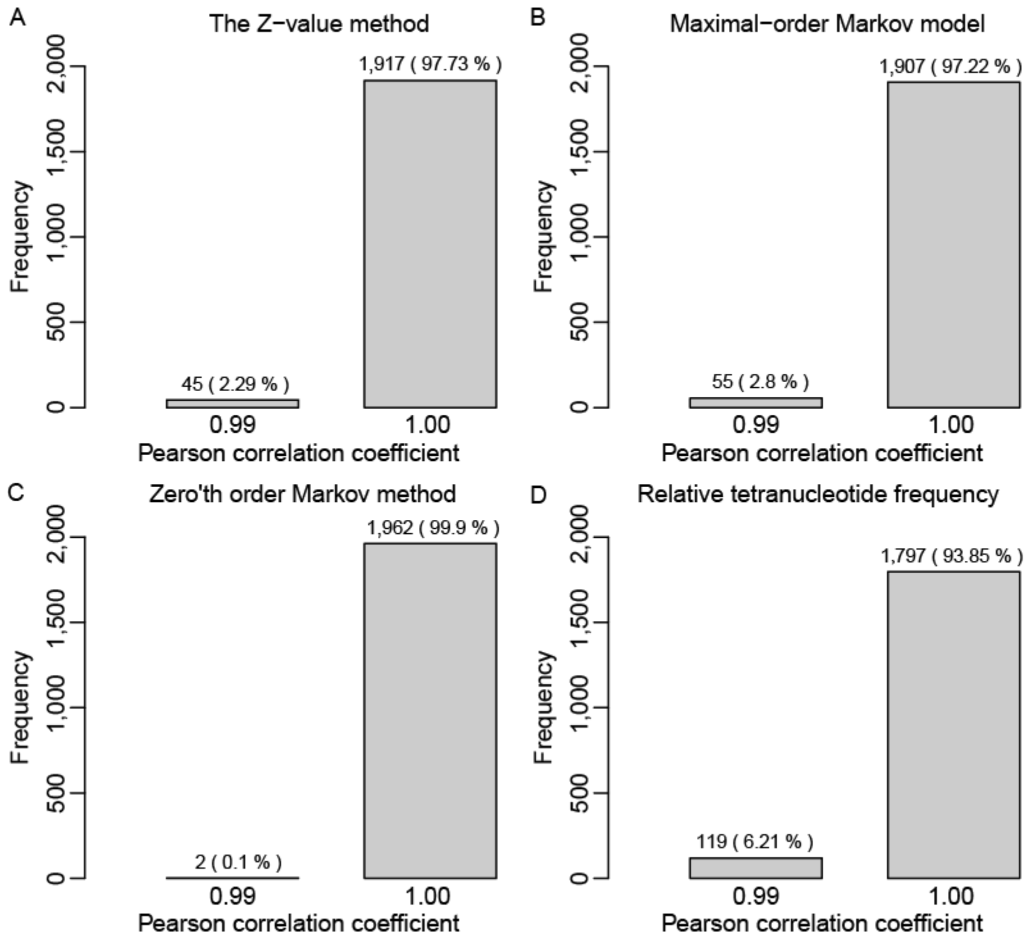

Figure S5. Intraspecific Pearson correlation coefficient distribution for each method. A, for the z-value method; B, for maximal-order Markov model; C, for zero-order Markov method; D, for relative tetranucleotide frequency. Intraspecific pairs with a Pearson correlation coefficient >0.99 cutoff from 1,964 intraspecific pairs were used for each method.

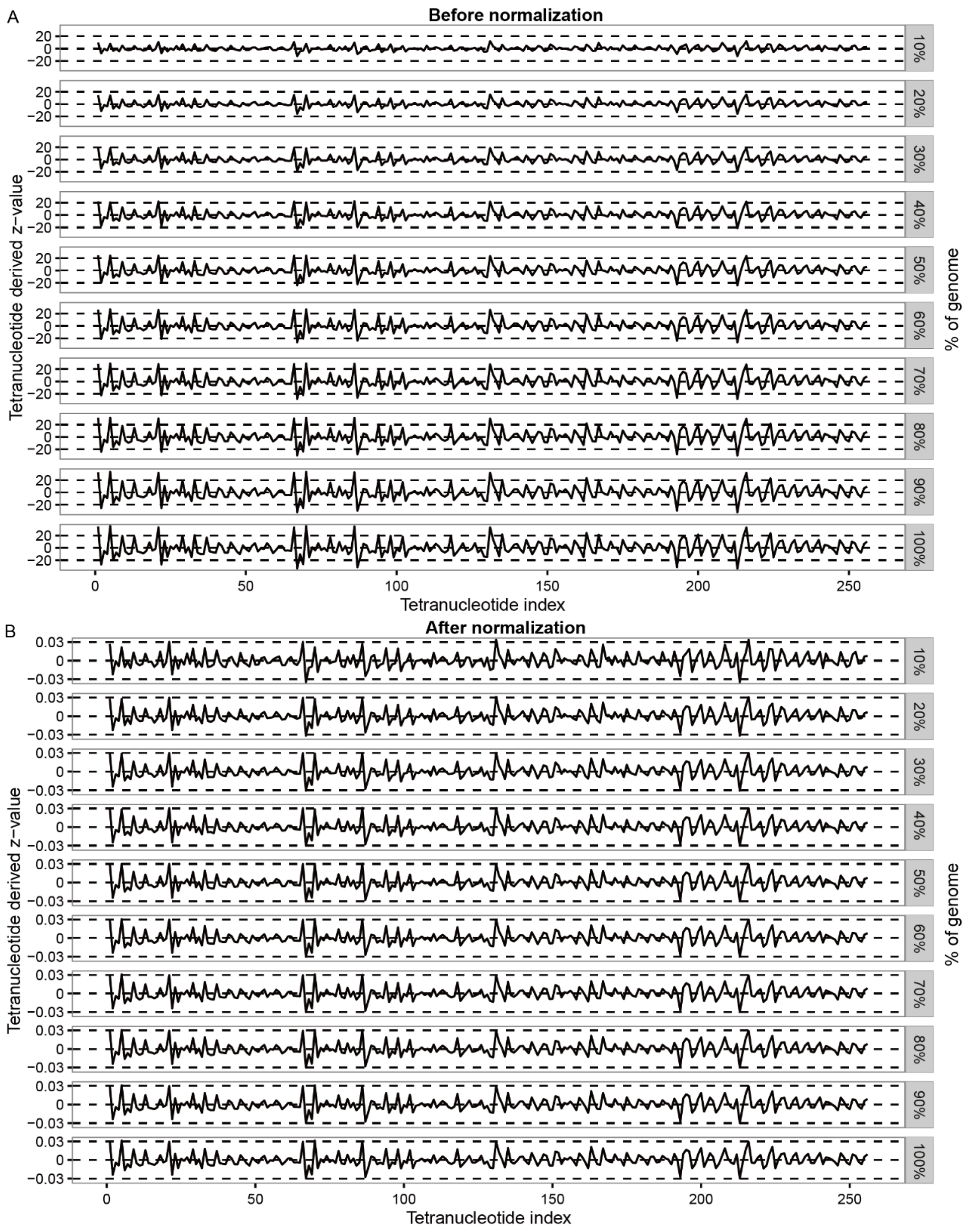

Figure S6. Normalization of tetranucleotide-derived z-values. A, before normalization; B, after normalization. These shown here represent the individual tetranucleotide deviations. The example shown here is for *Buchnera aphidicola* str. APS (*Acyrthosiphon pisum*) (GCA_000009605.1).

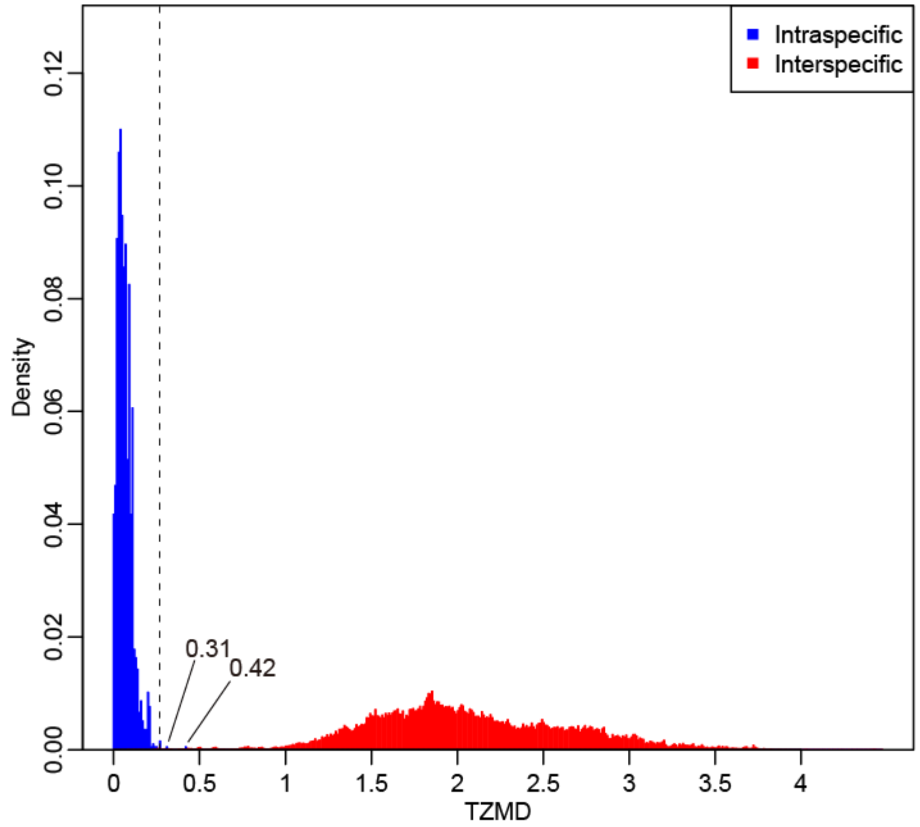

Figure S7. Histogram showing the TZMD distributions for both intraspecific and interspecific pairs. All values were generated using 1,779 queries (Additional file 1 Table S1) against 264 references (Additional file 1 Table S2), which comprise 1,964 intraspecific pairs and 467,692 interspecific pairs. *Borrelia hermsii* strains CC1 and HS1, and *Borreliella burgdorferi* strains CA382 and B31, which have an abnormally high TZMD of 0.31 and 0.42 respectively, are indicated.

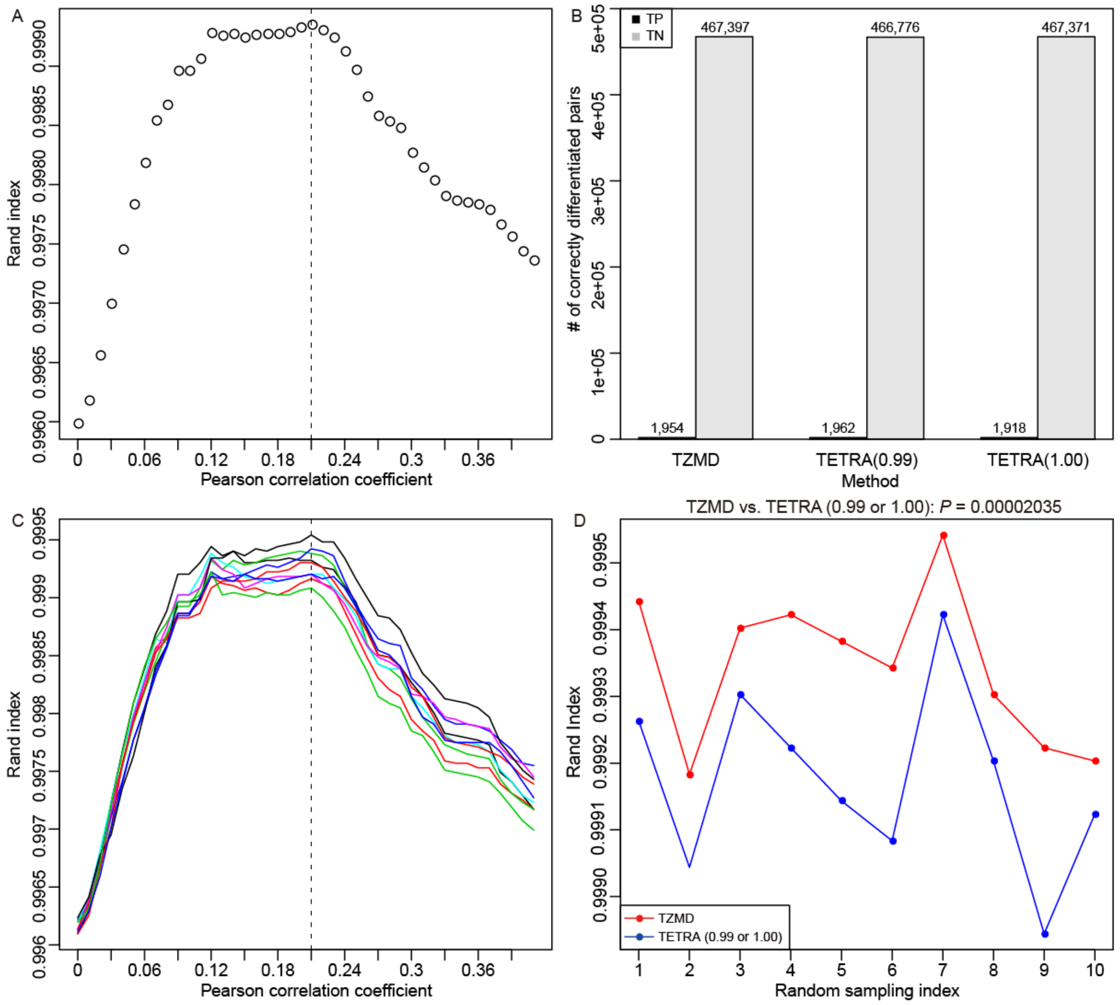

Figure S8. Comparison based on the Rand index showed that TZMD slightly improved species differentiation. (A) Determining the TZMD cutoff with the highest Rand index. (B) Bar plot showing the number of true positive (TP) and true negative (TN) for the two approaches. (C) The Rand indexes for 10 samplings for the TZMD method. (D) The highest Rand indexes for 10 samplings for both approaches. All 1779 queries against 264 references were used (panels A and B). For each sampling, 200 intraspecific and 50,000 interspecific pairs were randomly sampled (panels C and D). Because TETRA had two criteria (0.99 or 1.00) (Additional file 1 Figs. S1A and S3A), the TETRA method using both criteria were compared. Dashed line, the TZMD cutoff at 0.21; *P*-value, one-tailed paired t-test.

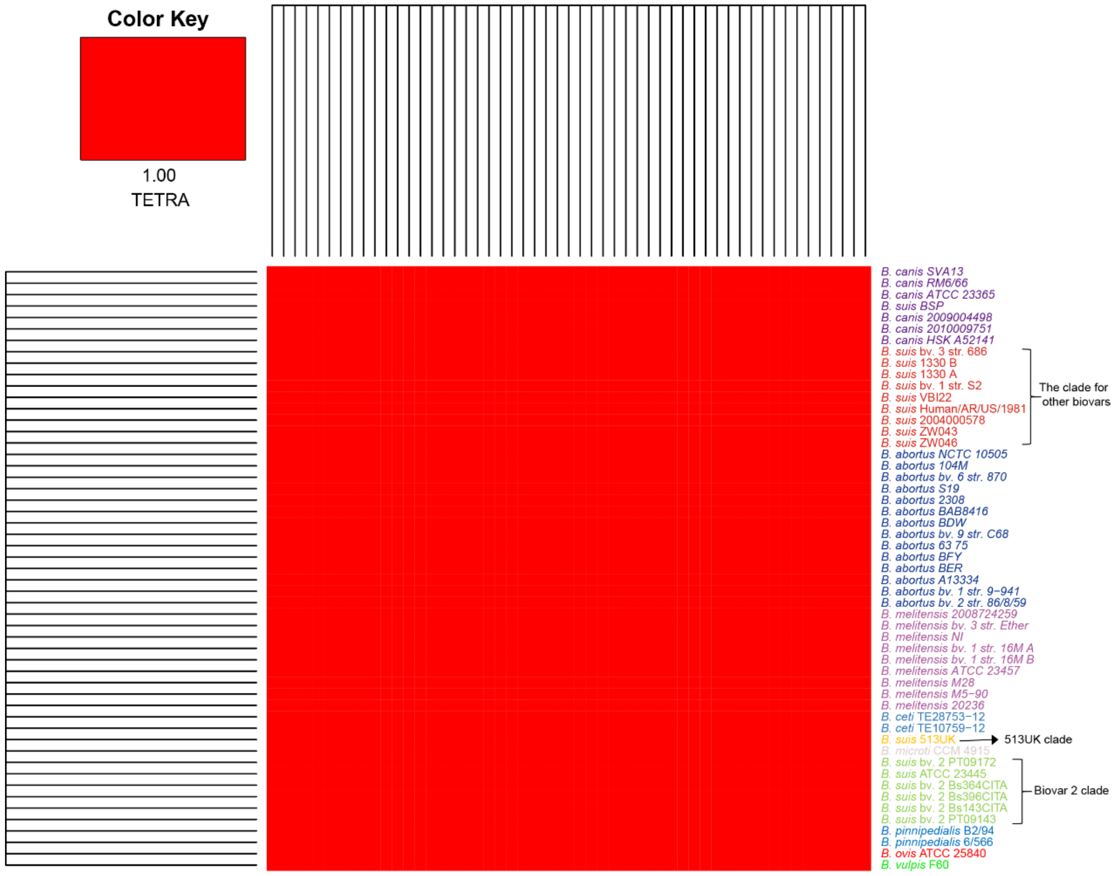

Figure S9. TETRA cannot differentiate *Brucella* species belonging to one single genospecies. TETRA value is used as a basis for color intensity. Different colors for species names indicates different clades. Three main clades for *B. suis* are also indicated. The figure was drawn by using the heatmap.2 function (gplots package, ward.D2 linkage).

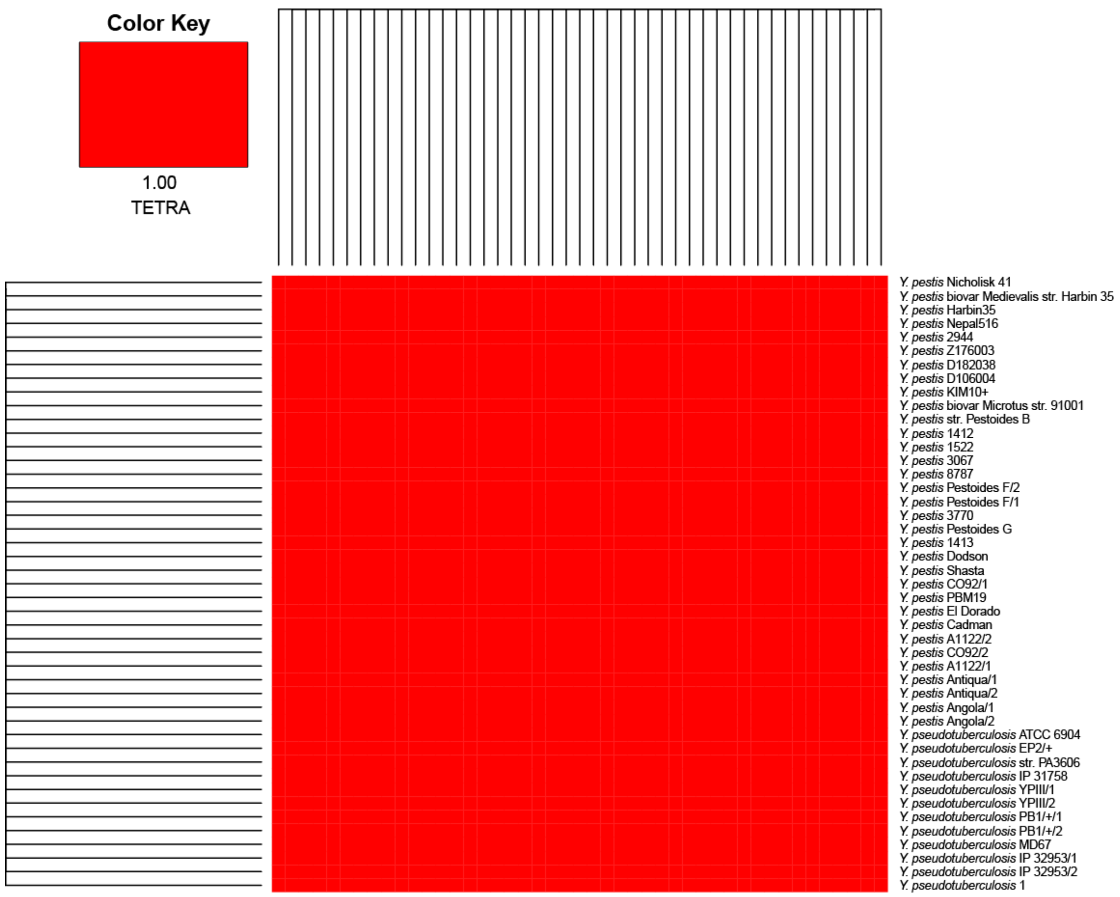

Figure S10. TETRA cannot differentiate *Y. pseudotuberculosis* and *Y. pestis* belonging to one single genospecies. TETRA value is used as a basis for color intensity. The figure was drawn by using the heatmap.2 function (gplots package, ward.D2 linkage).

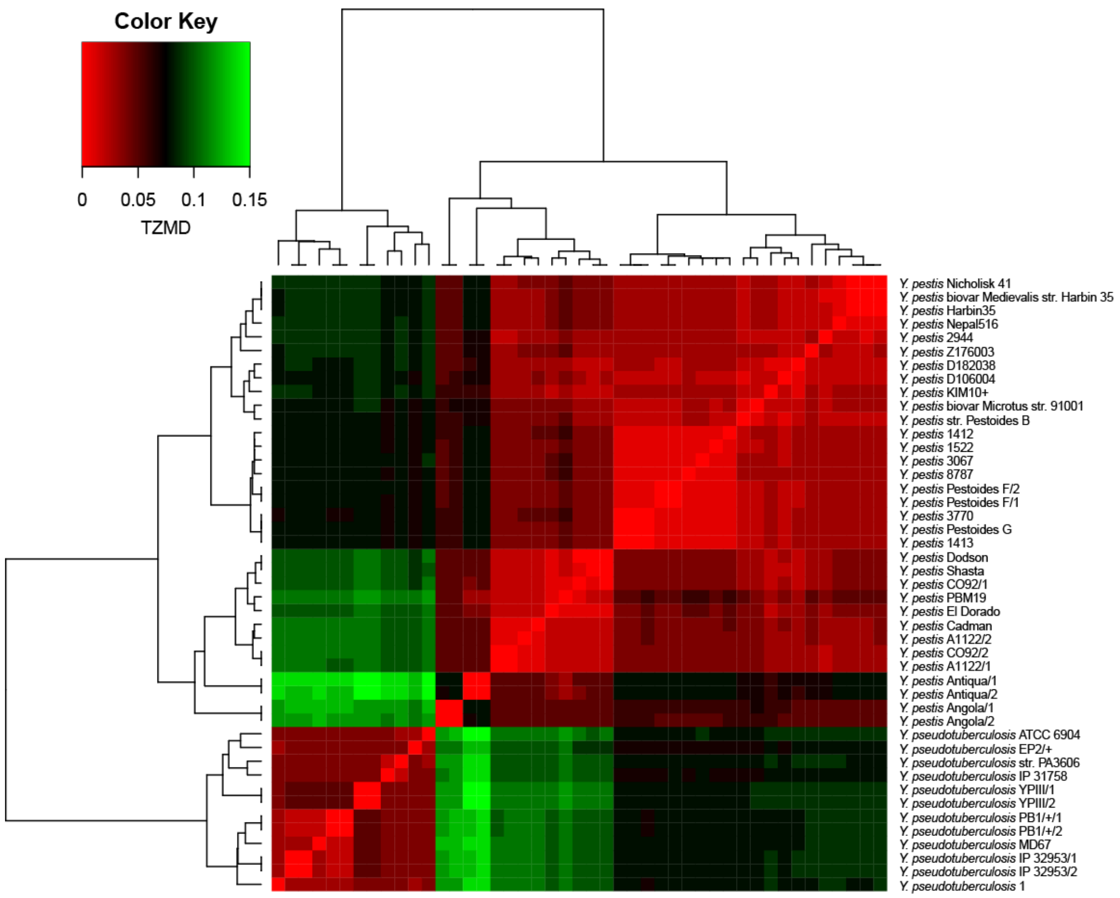

Figure S11. TZMD differentiates *Y. pestis* and *Y. pseudotuberculosis* belonging to one single genospecies. TZMD value is used as a basis for color intensity. The figure was drawn by using the heatmap.2 function (gplots package, ward.D2 linkage).

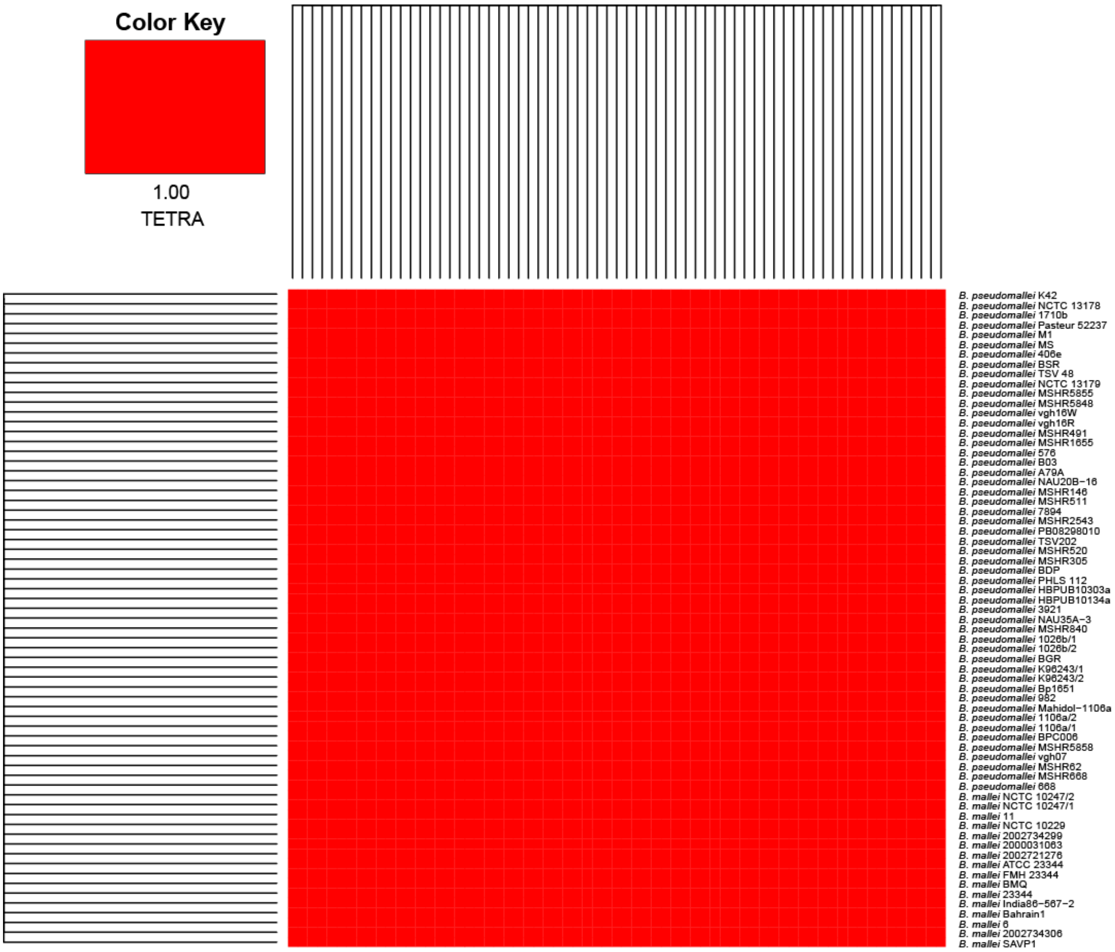

Figure S12. TETRA cannot differentiate *B. mallei* and *B. pseudomallei* belonging to one single genospecies. TETRA value is used as a basis for color intensity. The figure was drawn by using the heatmap.2 function (gplots package, ward.D2 linkage).

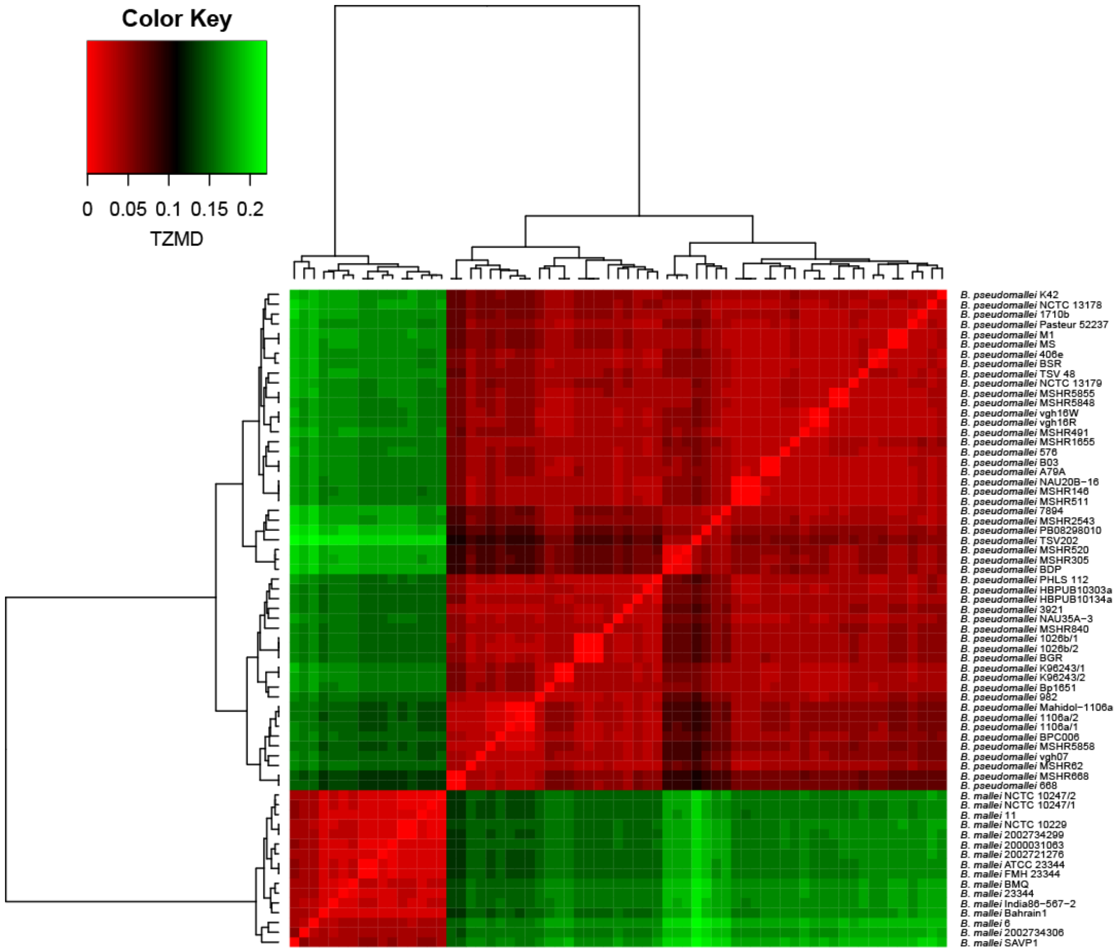

Figure S13. TZMD differentiates *B. mallei* and *B. pseudomallei* belonging to one single genospecies. TZMD value is used as a basis for color intensity. The figure was drawn by using the heatmap.2 function (gplots package, ward.D2 linkage).

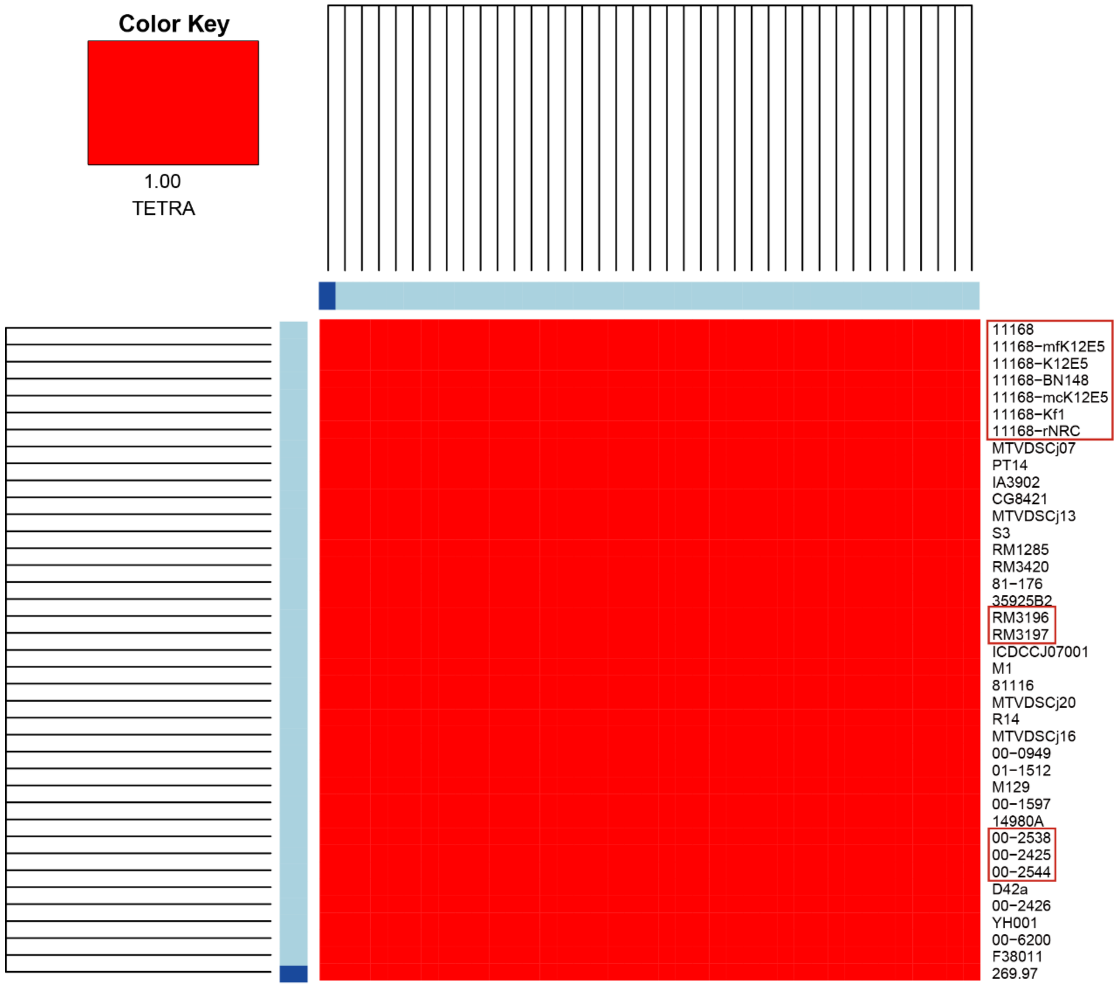

Figure S14. TETRA cannot differentiate subspecies and intraspecific strains of *Campylobacter jejuni*. TETRA value is used as a basis for color intensity. The boxed, clonal strains; blue bar, subsp. *doylei*; light blue bar, subsp. *jejuni*. The figure was drawn by using the heatmap.2 function (gplots package, ward.D2 linkage).

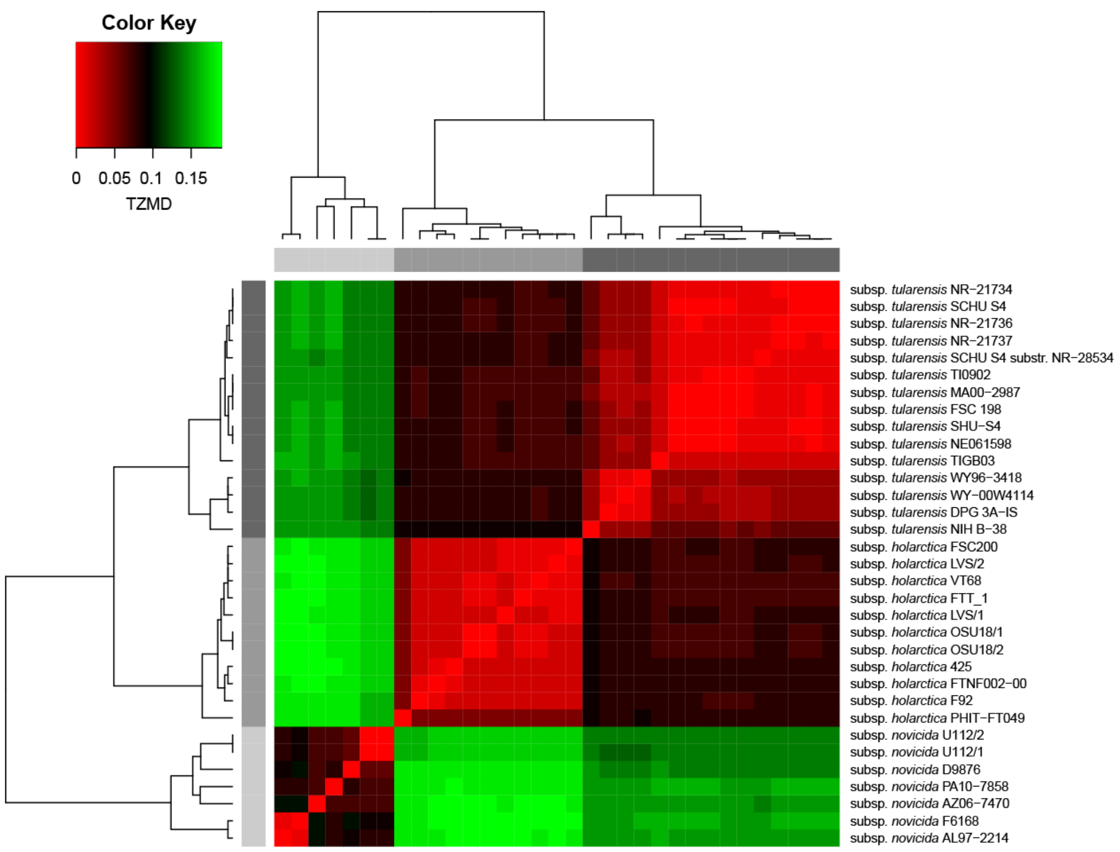

Figure S15. TZMD differentiates all subspecies of *Francisella tularensis*. TZMD value is used as a basis for color intensity. Light grey bar, subsp. *novicida*; medium grey bar, subsp. *holarctica*; dark grey bar, subsp. *tularensis*. The figure was drawn by using the heatmap.2 function (gplots package, ward.D2 linkage).

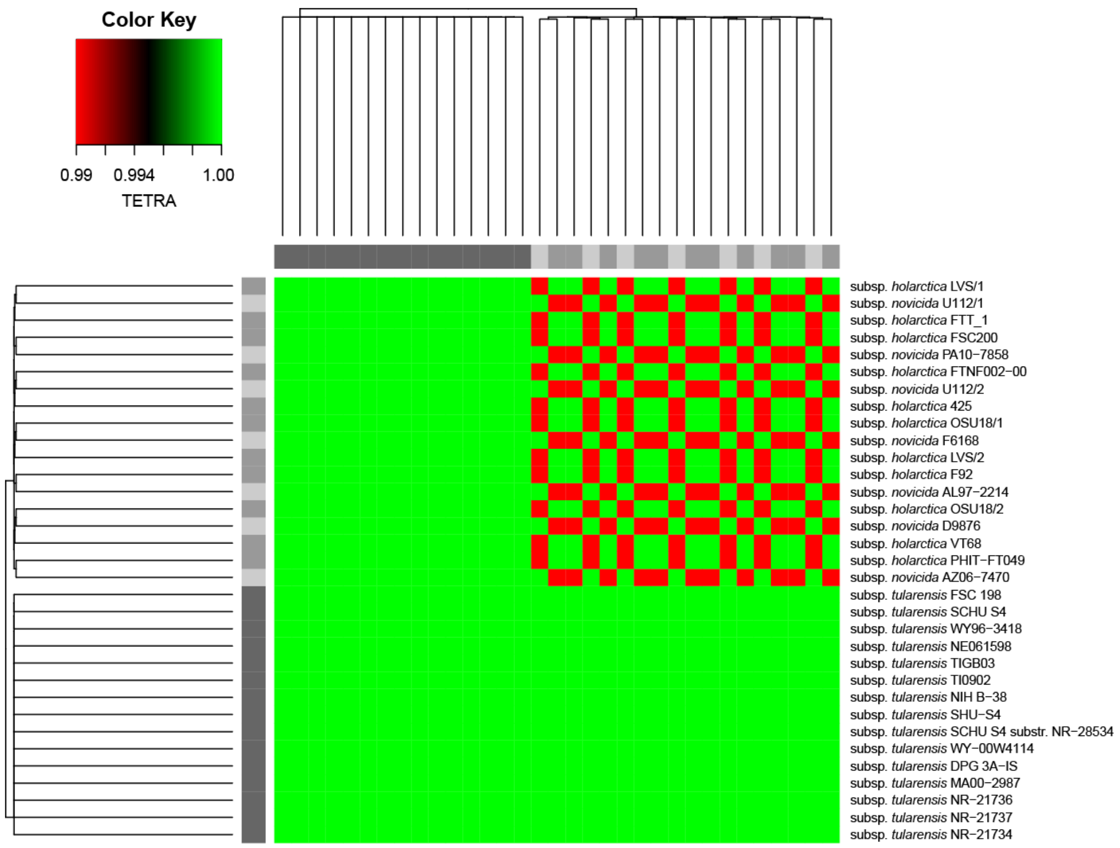

Figure S16. TETRA cannot differentiate two subspecies of *Francisella tularensis*. TETRA value is used as a basis for color intensity. Light grey bar, subsp. *novicida*; medium grey bar, subsp. *holarctica*; dark grey bar, subsp. *tularensis*. The figure was drawn by using the heatmap.2 function (gplots package, ward.D2 linkage).

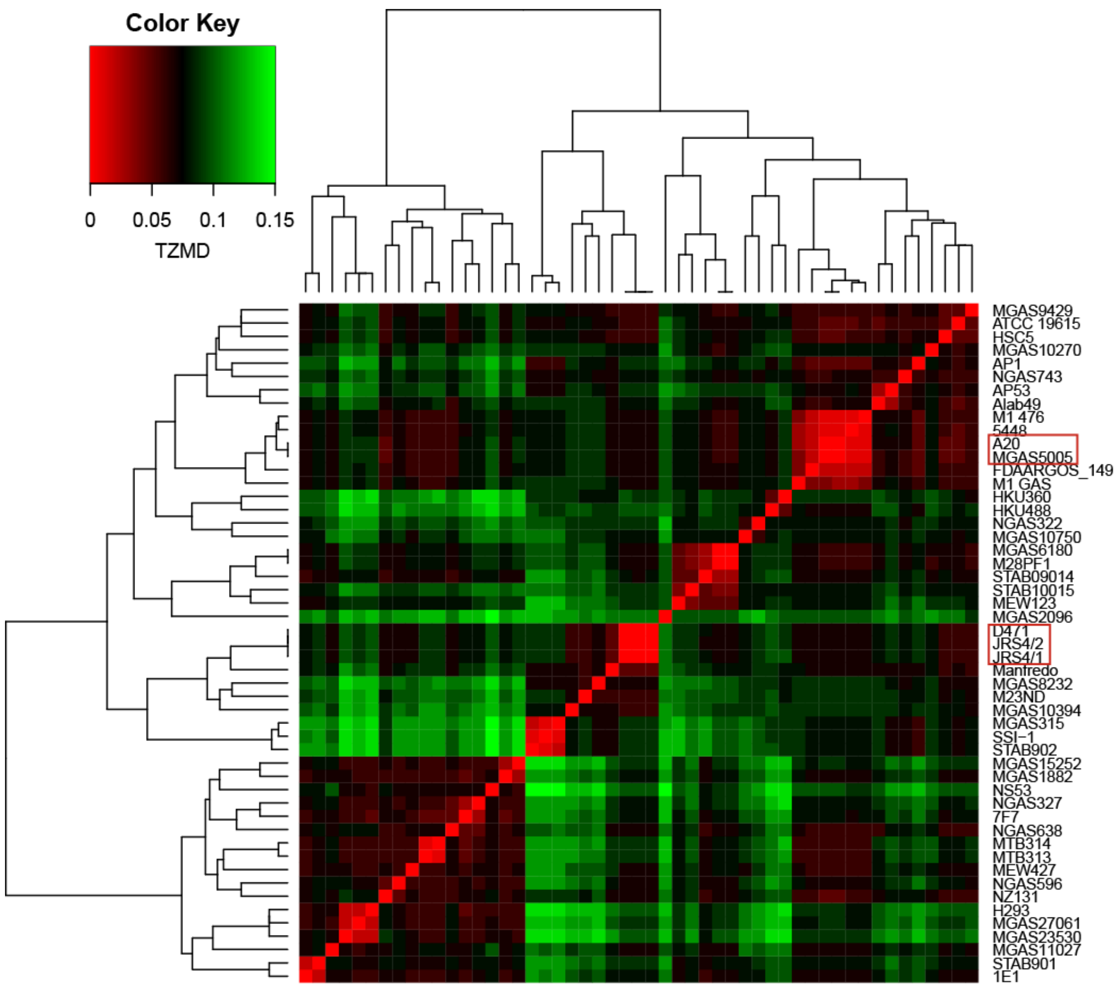

Figure S17. TZMD distinguishes intraspecific strains of *Streptococcus pyogenes*. TZMD value is used as a basis for color intensity. The boxed, clonal strains. The figure was drawn by using the heatmap.2 function (gplots package, ward.D2 linkage).

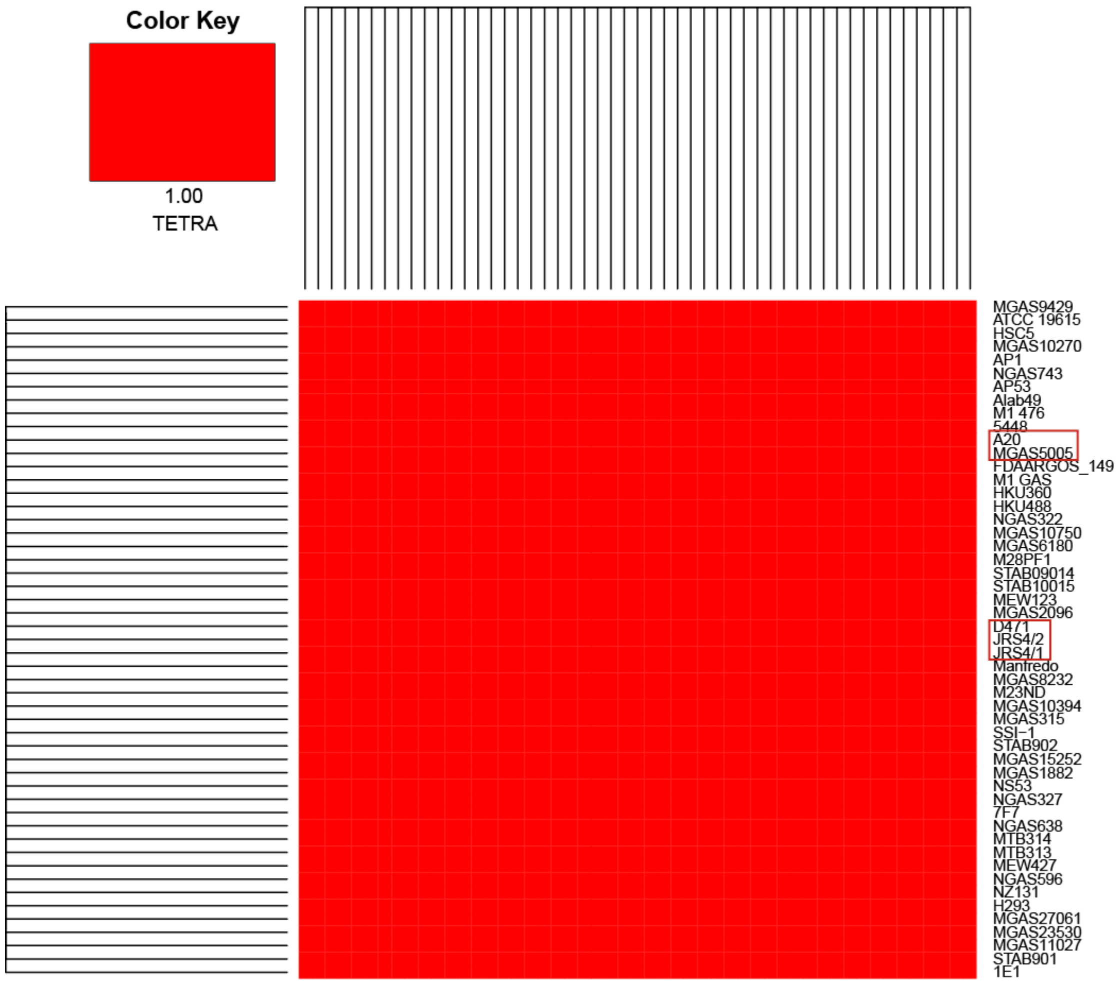

Figure S18. TETRA cannot differentiate intraspecific strains of *Streptococcus pyogenes*. TETRA value is used as a basis for color intensity. The boxed, clonal strains. The figure was drawn by using the heatmap.2 function (gplots package, ward.D2 linkage).

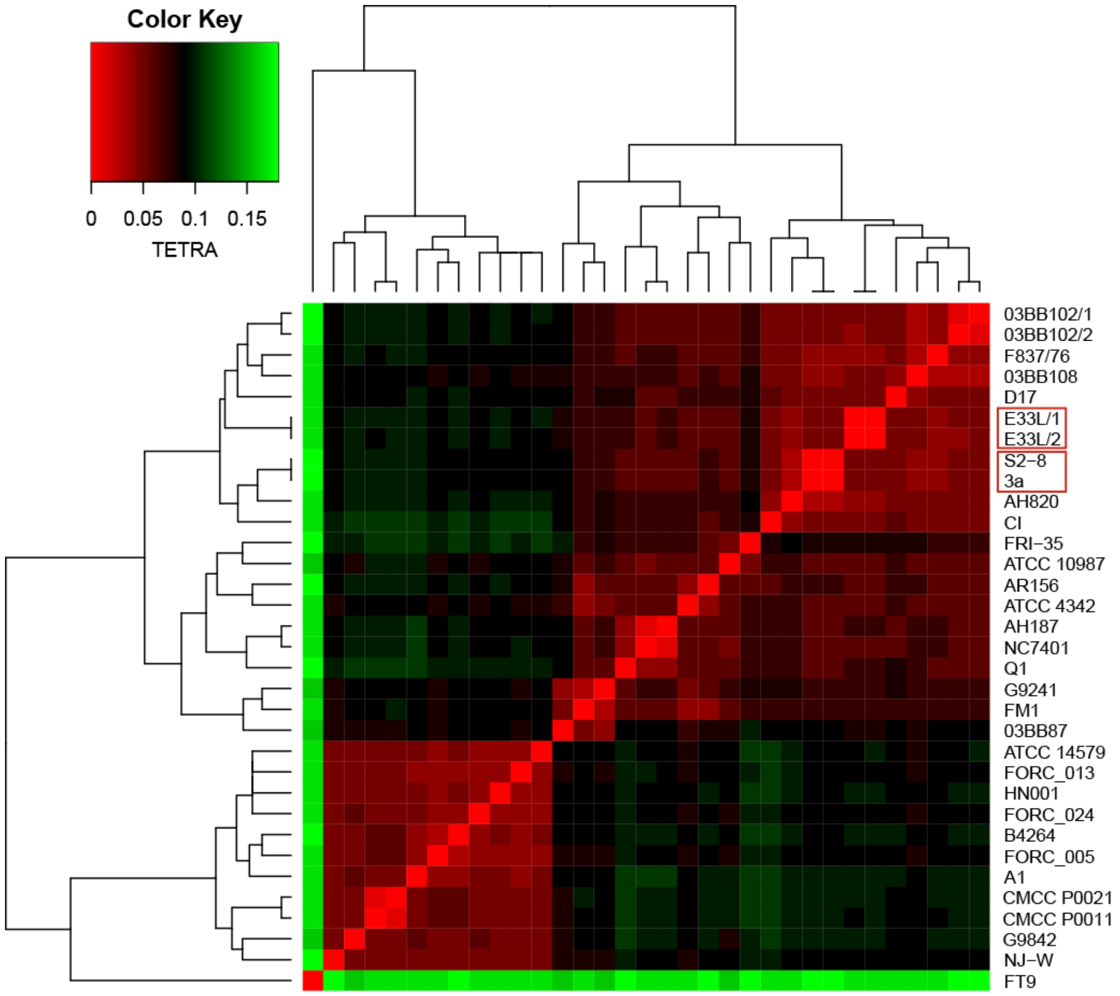

Figure S19. TZMD distinguishes intraspecific strains of *Bacillus cereus*. TZMD value is used as a basis for color intensity. The boxed, clonal strains. The figure was drawn by using the heatmap.2 function (gplots package, ward.D2 linkage).

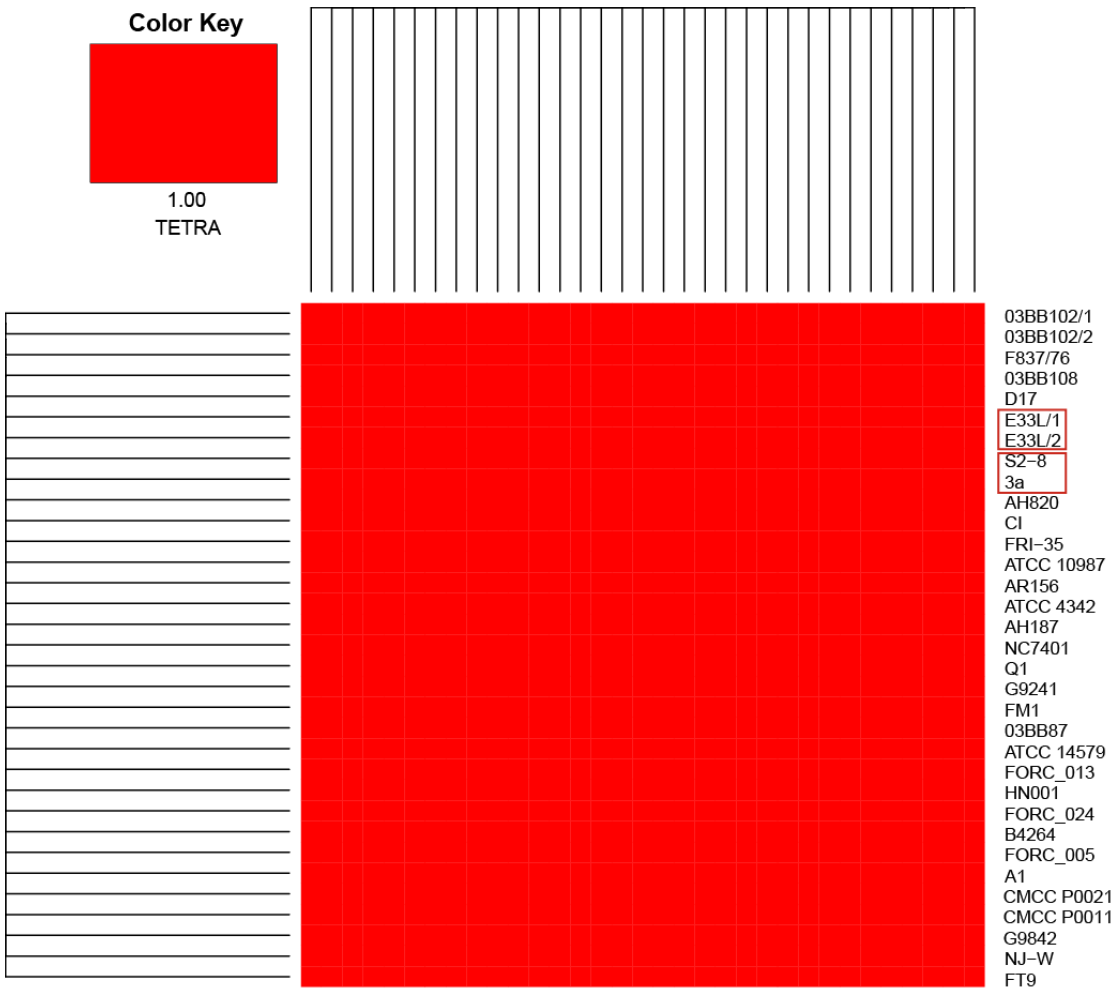

Figure S20. TETRA cannot differentiate intraspecific strains of *Bacillus cereus*. TETRA value is used as a basis for color intensity. The boxed, clonal strains. The figure was drawn by using the heatmap.2 function (gplots package, ward.D2 linkage).

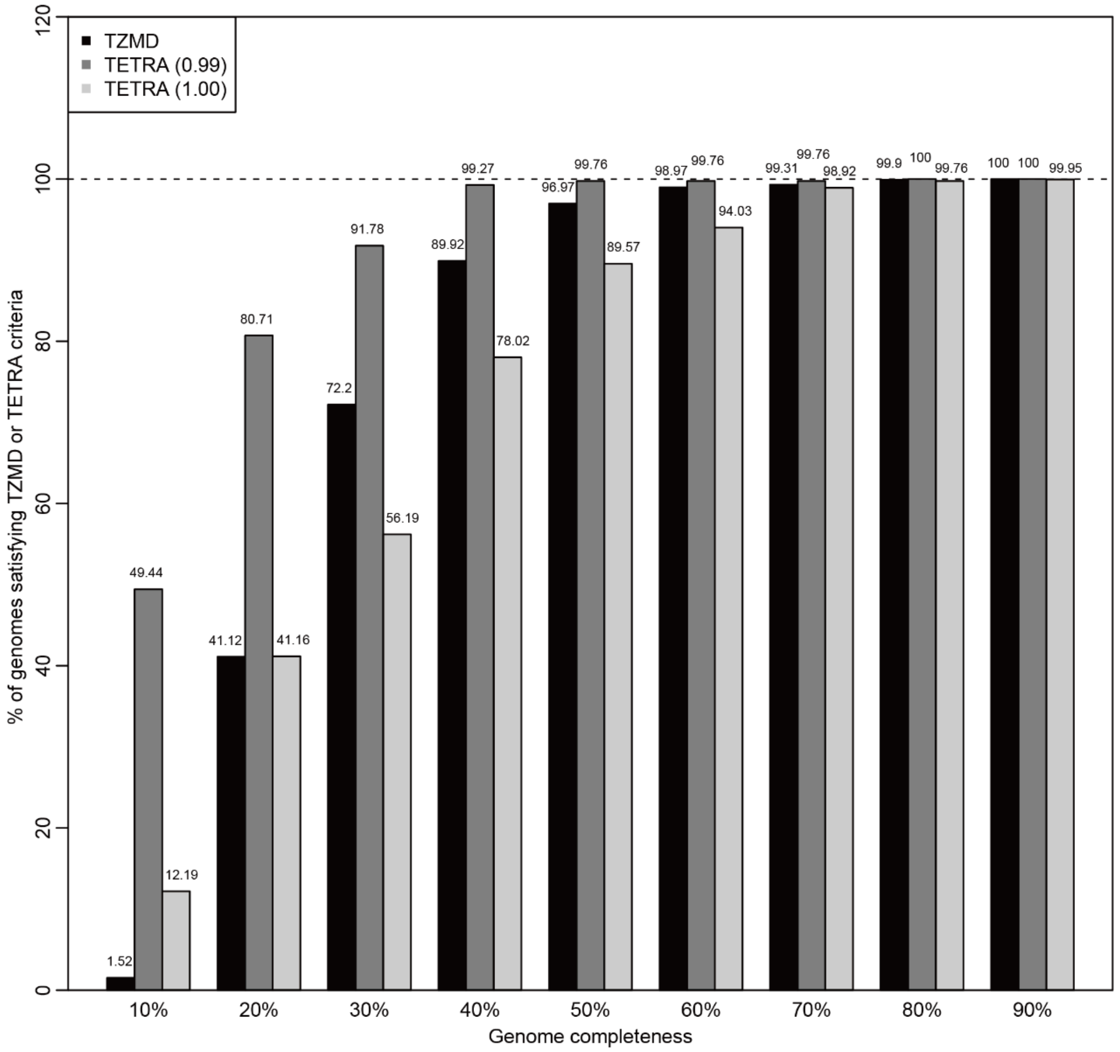

Figure S21. Impact of genomic completeness on TZMD and TETRA for species differentiation. TZMD cutoff, <0.21, TETRA cutoff, >0.99 or 1.00 (Supplementary material Fig. S1A). All 2,043 genomes (including 1,779 queries and 264 references) were summarized.

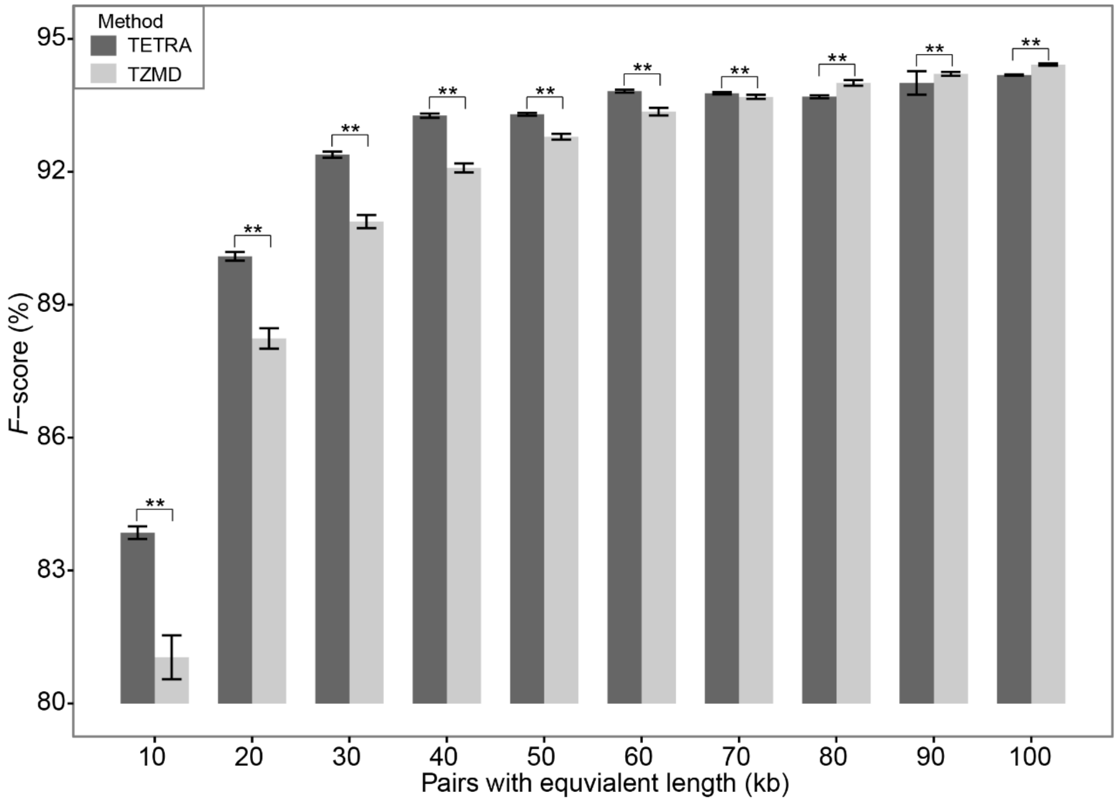

Figure S22. Binning performance of TZMD and TETRA. Binning performance is indicated by *F*-score. **, *P*-value <0.001, the paired t-test. A total of 1,779 queries and 264 references were sampled 10 times. For each sampling, ~320,000 intraspecific and 500,000 interspecific pairs 10 kb to 100 kb in length were sampled.

Table S1. TZMD and TETRA values for differently-sized genomes.

	Before normalization		After normalization	
**% of genome**	**TETRA**	**TZMD**	**TETRA**	**TZMD**
10	0.95	1587.4	0.95	0.74
20	0.98	1319.43	0.98	0.41
30	0.99	1091.84	0.99	0.29
40	0.99	896.03	0.99	0.24
50	1.00	738.61	1.00	0.20
60	1.00	575.68	1.00	0.16
70	1.00	412.76	1.00	0.12
80	1.00	279.3	1.00	0.10
90	1.00	135.2	1.00	0.06
100	1.00	0.00	1.00	0.00

Note: The shown here is for *Buchnera aphidicola* str. APS (*Acyrthosiphon pisum*) (GCA_000009605.1). The TETRA and TZMD are calculated for 10-100% of the genome against its full genome.
